# RE^(III)^ 3-Furoate Complexes: Synthesis, Structure, and Corrosion Inhibiting Properties

**DOI:** 10.3390/molecules27248836

**Published:** 2022-12-13

**Authors:** Vidushi P. Vithana, Zhifang Guo, Glen B. Deacon, Anthony E. Somers, Peter C. Junk

**Affiliations:** 1 College of Science & Engineering, James Cook University, Townsville, QLD 4811, Australia; 2 School of Chemistry, Monash University, Clayton, VIC 3800, Australia; 3 Institute for Frontier Materials, Deakin University, Burwood, VIC 3125, Australia

**Keywords:** rare earth metal, metathesis, carboxylate complexes, 3-furoates, structures, corrosion inhibitors

## Abstract

In this study, two types of Rare Earth (RE) 3-furoate complexes were synthesized by metathesis reactions between RE chlorides or nitrates and preformed sodium 3-furoate. Two different structural motifs were identified as Type **1RE** and Type **2RE**. The Type **1RE** monometallic complexes form 2D polymeric networks with the composition [RE(3fur)_3_(H_2_O)_2_]_n_ (**1RE** = 1La, 1Ce, 1Pr, 1Nd, 1Gd, 1Dy, 1Ho, 1Y; 3furH = 3-furoic acid) while Type **2RE** bimetallic complexes form 3D polymeric systems [NaRE(3fur)_4_]_n_ (**2RE** = 2Ho, 2Y, 2Er, 2Yb, 2Lu). The stoichiometric mole ratio used (RE: Na(3fur) = 1:3 or 1:4) in the metathesis reaction determines whether **1RE** or **2RE** (RE = Ho or Y) is formed, but **2RE** (RE = Er, Yb, Lu) were obtained regardless of the ratio. The corrosion inhibition behaviour of the compounds has been examined using immersion studies and electrochemical measurements on AS1020 mild steel surfaces by a 0.01 M NaCl medium. Immersion test results revealed that [Y(3fur)_3_(H_2_O)_2_]_n_ has the highest corrosion inhibition capability with 90% resistance after 168 h of immersion. Potentiodynamic polarisation (PP) measurements also indicate the dominant behaviour of the **1Y** compound, and the PP curves show that these rare earth carboxylate compounds act predominantly as anodic inhibitors.

## 1. Introduction

Mild steel is extensively used as an engineering material in diverse industries due to its relatively low cost, high machinability, and widespread availability [1]. However, these kinds of metals and alloys are vulnerable to corrosion degradation by chemical and/or electrochemical reactions [2,3]. A range of factors such as pressure, temperature, and aggressive acidic environmental conditions [3] can lead to premature failure in mild steel structures. Over 3% of the world’s Gross Domestic Production equating to US $2.5 trillion is spent annually to replace and reduce the damage caused by corrosion [4,5]. A major part of this cost includes the maintenance and replacement of water recirculating pipelines, bridges, and water tanks associated with cooling systems [3,6]. Without a proper control method to mitigate corrosion, such assets would have a significantly limited lifetime [7]. The latest reports by NACE international’s IMPACT researchers have revealed that the application of currently available corrosion control practices could save between US $375 and US $875 billion annually. Thus 15–35% of the global cost of corrosion could be saved [5]. To protect metals and alloys from corrosion, a range of approaches are used. Among them, one of the oldest and most cost-efficient protection methods is using corrosion inhibitors [8,9]. Corrosion inhibitors are widely incorporated in water treatment formulations, as pigments in paint coatings, pipeline streams, etc. [8,10]. Normally, these inhibitors make a protective barrier film on the metal surface to minimize corrosion attacks [11].

Among corrosion inhibitors, rare earth (RE) metal salts have been recognized as an effective and environmentally friendly nontoxic method to prevent corrosion [12,13,14,15,16,17]. The use of rare earths as corrosion inhibitors has emerged as an alternative to replace hexavalent chromates, some of the most extensively used corrosion inhibitors, and their high toxicity and carcinogenic properties have led to a significant reduction in their use in nearly all cases [12,18].

Moreover, organic systems containing hetero atoms are considered to have an advanced inhibiting ability due to the strong interactions between N, O and S atoms and the metallic surfaces [19,20,21,22]. Although there are many studies of heterocyclic compounds as corrosion inhibitors for mild steel, only a limited amount of literature is reported on furan and its derivatives for such applications. Vaidyanathan and Hackerman investigated the structural effect of some furan derivatives on corrosion inhibition and the effect on the dissolution of iron. The inhibition efficiency of the tested compounds follows the order 2-furoic acid > furfurylamine > tetrahydrofurfurylamine. The inhibition ability of furoic acid was explained based on the electron-donating ability of the heterocyclic ring oxygen as well as the carboxylic oxygen [23]. Khaled tested three furan derivatives (methyl 2-furoate, ethyl 2-furoate, and amyl 2-furoate) on mild steel in 1M HCl to evaluate the anti-corrosive properties with respect to the changes in substituent and polarity of the compound. It was suggested that the protection of mild steel occurs through donating to or sharing electrons with the metal surface, forming a stable covalent coordination bond (chemisorption) [24].

The recent innovative approaches toward designing “Green” corrosion inhibitors have involved combining RE ions with an organic ligand to form new complexes with mixed inhibition functions [10,15,16]. RE-carboxylate complexes are readily formed due to the highly oxophilic nature of REs towards carboxylate ligands and they tend to adopt either dimeric or polymeric forms [25,26]. Over the years, several different coordination patterns have been observed with carboxylate ligands binding to metal atoms [25,27].

So far, several trivalent rare-earth carboxylates have been synthesized and evaluated for their corrosion inhibition properties on mild steel [7,10,15,16,28,29]. Some of the best-performing RE carboxylate complexes reported in recent years are cerium salicylate [10] lanthanum 4-hydroxycinnamate [30] and yttrium 3-(4′-methylbenzoyl)propionate [11]. The ligands feature an additional donor function, which may enable binding (bridging) to a steel surface. [10,15,16]. Our latest paper evaluated the inhibition properties of thiophene derivatives on mild steel by investigating the rare earth metal complexation by 3-thiophenecarboxylate complexes [31], where the S atom provides an opportunity for bridging to steel.

**Chart 1:**
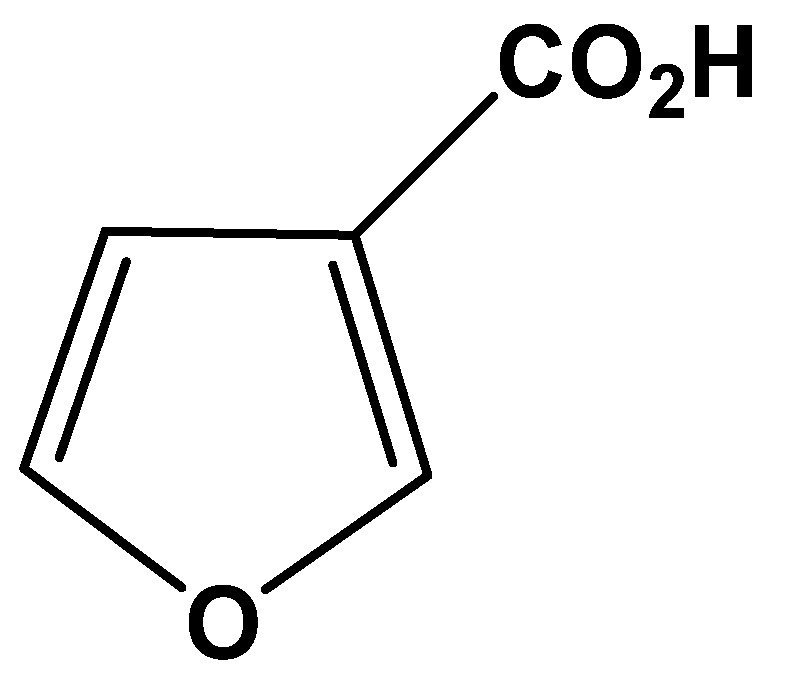
Structure of 3-furoic acid (3FurH).

In this paper, we discuss the synthesis of RE 3-furoate complexes (3furH = 3-furoic acid, Chart 1) and the corrosion inhibitory properties for AS 1020 mild steel in 0.01 M NaCl corrosive media, to examine whether the oxygen donor atom might prove more efficacious than sulfur. The synthesized compounds were also characterized using several techniques including single crystal X-ray crystallography. Moreover, the corrosion inhibition properties of the synthesized compounds were determined by weight loss experiments combined with potentiodynamic polarization measurements.

## 2. Results

### 2.1. Synthesis and Characterization

**Scheme 1:**
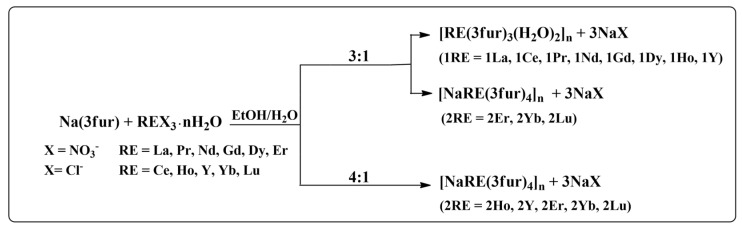
The synthesis of RE 3-furoate complexes. The ratios shown in the Scheme refer to the Na(3fur): REX_3_ ratios of reactants.

When the syntheses were carried out with a ligand: RE stoichiometry of 3:1, eight homometallic complexes [RE(3fur)_3_(H_2_O)_2_]_n_ (**1RE** = 1La, 1Ce, 1Pr, 1Nd, 1Gd, 1Dy, 1Ho, 1Y), as well as Na-RE bimetallic complexes [NaRE(3fur)_4_]_n_ (**2RE** = 2Er, 2Yb, 2Lu), were obtained. Upon treating the ligand and RE salts in a 4:1 mole ratio, heterobimetallic complexes [NaHo(3fur)_4_]_n_ (**2Ho**) and [NaY(3fur)_4_]_n_ (**2Y**) were obtained in addition to the complexes **2RE** = 2Er, 2Yb and 2Lu, but La to Dy still gave [RE(3fur)_3_(H_2_O)_2_]_n_. Thus, the ratio used in the metathesis reaction appeared to affect the identity of the final complex for holmium and yttrium (Scheme 1).

**Figure 1:**
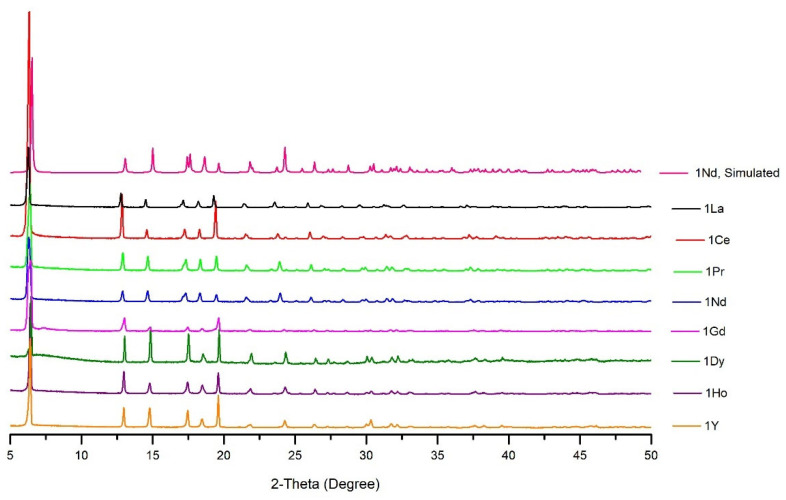
PXRD traces of the [RE(3fur)_3_(H_2_O)_2_]_n_ **1RE** series compared to the simulated pattern of [Nd(3fur)_3_(H_2_O)_2_]_n_ (**1Nd**).

**Figure 2:**
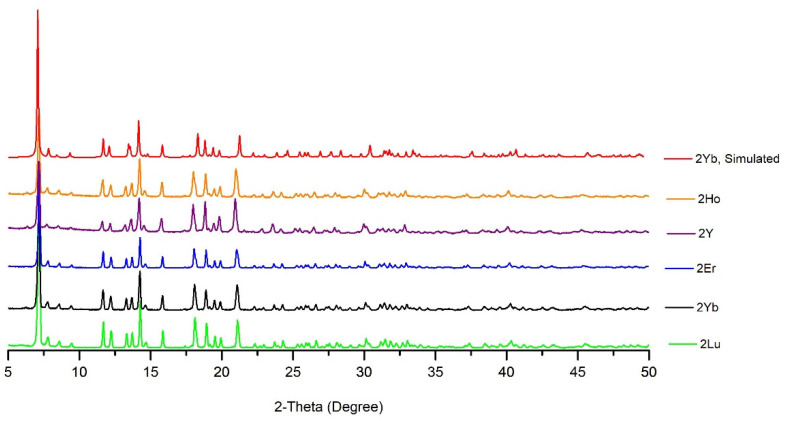
PXRD traces of the [NaRE(3fur)_4_]_n_ **2RE** series compared to the simulated pattern of [NaYb(3fur)_4_]_n_ (**2Yb**).

In all cases, single crystals of the compounds were obtained by slow evaporation of the mother liquor. The bulk samples of complexes [RE(3fur)_3_(H_2_O)_2_]_n_ (**1RE** = 1La, 1Ce, 1Pr, 1Nd, 1Gd, 1Dy, 1Ho, 1Y) (Figure 1) and [NaRE(3fur)_4_]_n_ (**2RE** = 2Ho, 2Y, 2Er, 2Yb, 2Lu) (Figure 2) gave X-ray powder diffraction patterns that are identical to each other and are in agreement with the patterns generated from the single-crystal data.

Satisfactory elemental analyses were obtained for all the compounds, with the exception of compound **2Yb**, with a low % C value. This is a frequent issue recognized for rare earth and alkaline earth complexes within previous literature [32,33,34,35,36,37,38]. However, X-ray crystallographic data together with additional % RE analyses and TGA data offer further evidence to confirm the final chemical compositions of the complexes.

The IR spectra of [RE(3fur)_3_(H_2_O)_2_]_n_ (**1RE** = 1La, 1Ce, 1Pr, 1Nd, 1Gd, 1Dy, 1Ho, 1Y) (Figure S1) and [NaRE(3fur)_4_]_n_ (**2RE** = 2Ho, 2Y, 2Er, 2Yb, 2Lu) (Figure S2) show strong similarities in major bands within each isostructural series. Thus, the significant IR bands for only one compound from each series (**1Nd** and **2Yb**) are listed in Table 1. For the **1RE** compounds broad O-H stretching bands in the range of 3200–3550 cm^−1^ are comparable and attributed to the presence of coordinated water molecules. IR spectra of all the RE complexes show strong asymmetric and symmetric carboxylate stretching bands at 1530–1350 cm^−1^. Relatively small Δν(CO_2_^−^) values are indicative of the presence of chelating and/or bridging carboxylate ligands [39].

A TGA study was conducted to establish the thermal behaviour of the complexes as well as the intermediates and products formed during the thermolysis. TGA curves of the **1RE** bulk sample (Figure S3) showed a steep initial weight loss (*ca*. 75–130 ℃), which corresponds to the loss of two molecules of water and the results are in agreement with the composition determined crystallographically as [RE(3fur)_3_(H_2_O)_2_]_n_. In line with the single-crystal compositions of the **2RE** compounds, their thermograms (Figure S4) do not indicate any weight loss owing to the loss of coordinated or lattice solvent molecules. Further weight loss is observed for all the compounds at ca. 370–500 ℃. However, the TGA plot of **1La** exhibits significantly different behaviour as the product formation starts and finishes at lower temperatures than for the rest of the compounds in the isostructural series. The products plausibly resulted from the decomposition of the anhydrous complexes to the RE_2_(CO_3_)_3_ in the **1RE** series and Na_3_RE(CO_3_)_3_ in the **2RE** series. The IR spectra of the residues of compounds **1Ce** and **2Yb** after thermolysis to 500 ℃ support this interpretation [40,41] since they correspond well with the previously reported spectrum of [Dy_2_(CO_3_)_3_].4H_2_O (Figure S5) [42]. However, for the residues of [RE(3fur)_3_(H_2_O)_2_]_n_ compounds, there is not sufficient weight loss for RE_2_(CO_3_)_3_ alone. Residual 3-furoate ligand could not be detected in the residue by IR spectra, so there must be an amorphous or IR silent coproduct such as RE(OH)_3_, RE_2_O_3_, or REC_2_. Metal carboxylates have a number of possible decomposition paths including the formation of carbonates and oxides [43,44].

Liquid chromatography-mass spectrometry (LCMS) studies have been used to investigate the composition of complexes **1Dy**, **2Ho** and **2Lu** in solution. In the mass spectrum of monometallic complex **1Dy** the molecular ion peak [Dy(3fur)_2_(H_2_O)_2_]^+^ can be detected at m/z 422.1 with the correct isotope patterns. Other than that, the related ion such as [Dy(3fur)(OH)EtOH]^+^, can be also observed. The bimetallic complexes **2Ho** and **2Lu** were also examined by LCMS. In the positive mode of complex **2Ho**, [Ho_2_(3fur)_4_(HCO_3_)(H_2_O)]^+^ and [Na_2_Ho(3fur)_3_(3furH)(HCO_3_)(H_2_O)]^+^ were detected at m/z 853.5 and 735.2, respectively, with low intensity. Finally, compound **2Lu** in LCMS led to a highly intense peak as [Na_2_(CO_3_)Lu(3fur)_4_(H_2_O)_2_]^-^ in the negative mode at m/z 761. Interestingly, mass spectrometry results suggest that the bimetallic complexes remain considerably intact in solution. Furthermore, the observations of LCMS experiments have revealed that all the tested complexes retain some form of rare earth carboxylate fragments in solution.

### 2.2. X-ray Crystallography

**Figure 3:**
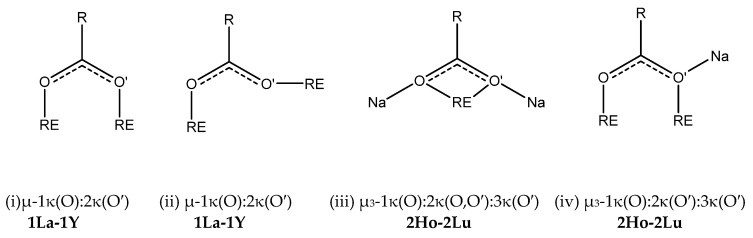
Different coordination modes observed for carboxylate ligand (3-furoate) in rare earth complexes. (i): *syn*-*syn* (ii): *syn*-*anti*.

There are three distinct carboxylate ligand binding modes, namely bridging µ-1κ(O):2κ(O′), chelating double bridging µ_3_-1κ(O):2κ(O,O′):3κ(O′) and double bridging µ_3_-1κ(O):2κ(O′):3κ(O′) observed in the structures (Figure 3).

#### 2.2.1. Monometallic 2D Polymeric Chains of [RE(3fur)_3_(H_2_O)_2_]_n_ Complexes (1RE = 1La, 1Ce, 1Pr, 1Nd, 1Gd, 1Dy, 1Ho, 1Y)

**Figure 4:**
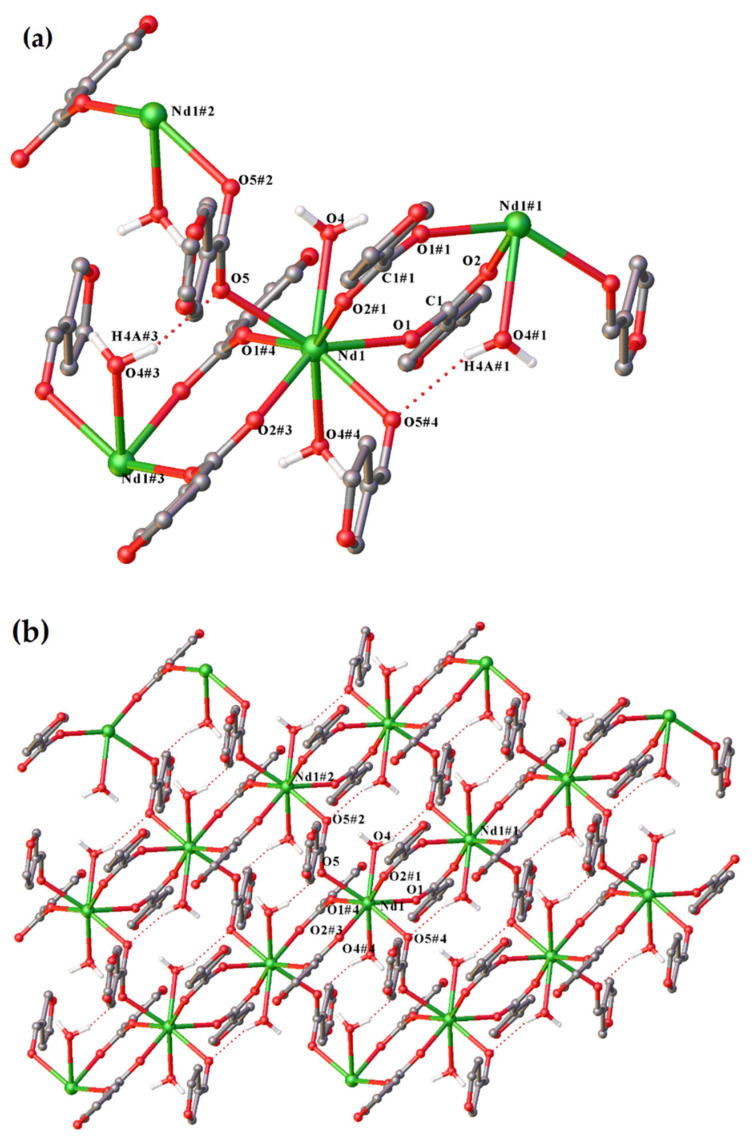
(**a**) The immediate coordination environment around Nd metal atoms in [Nd(3fur)_3_(H_2_O)_2_]_n_ (**1Nd**); (**b**) 2D polymeric chain of **1Nd** (representative of **1RE** = 1La, 1Ce, 1Pr, 1Gd, 1Dy, 1Ho, 1Y) Selected bond lengths (Å) and angles (°) (Data for all **1RE** in Supplementary information; Tables S2 and S3). **1Nd** Nd1-O1#4 2.3487(15), Nd1-O1 2.3487(15), Nd1-O2#1 2.3991(13), Nd1-O2#3 2.3991(13), Nd1-O5#4 2.4848(14), Nd1-O5 2.4848(14), Nd1-O4#4 2.5830(14), Nd1-O4 2.5830(14), Nd1…Nd1#1 5.1246(18), Nd1#1-Nd1-Nd1#3 167.339(5), O4-Nd-O4#4 127.16(6), Symmetry Code: ^#1^ 1/2-X,3/2-Y,-Z; ^#2^ 1-X,+Y,3/2-Z; ^#3^ 1/2+X,3/2-Y,1/2+Z:, ^#4^ 1-X,+Y,1/2-Z.

This isomorphous series of complexes crystallizes in the monoclinic *C*2/*c* space group and the compounds have 2D polymeric structures with eight-coordinate rare earth metal atoms with distorted square antiprismatic geometry. Crystal refinement data and bond lengths are provided in the Supplementary Material (Tables S1 and S2). A representative structure of [Nd(3fur)_3_(H_2_O)_2_]_n_ is shown in Figure 4. The coordination environment comprises of six bridging µ-1κ(O):2κ(O’) carboxylate ligands binding through O(1, 1#4, 2#1, 2#3, 5, 5#4) and two ligated aqua molecules O(4, 4#4). The backbone of the 1D polymeric chain is constructed through eight-membered rings (Nd1-O2#1-C1#1-O1#1-Nd1#1-O2-C1-O1) formed by *syn-syn* bridging bidentate carboxylates between adjacent Nd1 and Nd1#1 metal centres. The 1D polymeric chain of Nd atoms is nonlinear with Nd1#1-Nd1-Nd1#3 at an angle of 167.339(5)° and the distance between the adjacent Nd^3+^ metal centres in the chain is 5.1246(18) Å. The two ligated water oxygen atoms form a O4-Nd1-O4#4 *cisoid* angle of 127.16(6)°. Two of the bridging carboxylate oxygen atoms (O5 and O5#4) are involved in hydrogen bonding to water oxygen atoms (O4#3 and O4#1) attached to the adjacent metal centres. The 1D polymeric chain propagates into a 2D network by *syn-anti* bridging bidentate ligands (O5, 5#2) between Nd1 and Nd1#2 metal atoms. Experimentally found H bonds of the compound [Nd(3fur)_3_(H_2_O)_2_]_n_ are shown in Figure 4a and H bond lengths are listed in Table S4. Lanthanoid contraction [45] is observed across the period from La to Y with a decrease in the average RE-O bond distance (Table S2) [RE = La 2.512 Å, Ce 2.487 Å, Pr 2.470 Å, Nd 2.454 Å, Gd 2.404 Å, Dy 2.379 Å, Ho 2.365 Å, Y 2.365 Å].

#### 2.2.2. Bimetallic 3D Polymeric Chains of [NaRE(3fur)_4_]_n_ Complexes (2RE = 2Ho, 2Y, 2Er, 2Yb, 2Lu)

**Figure 5:**
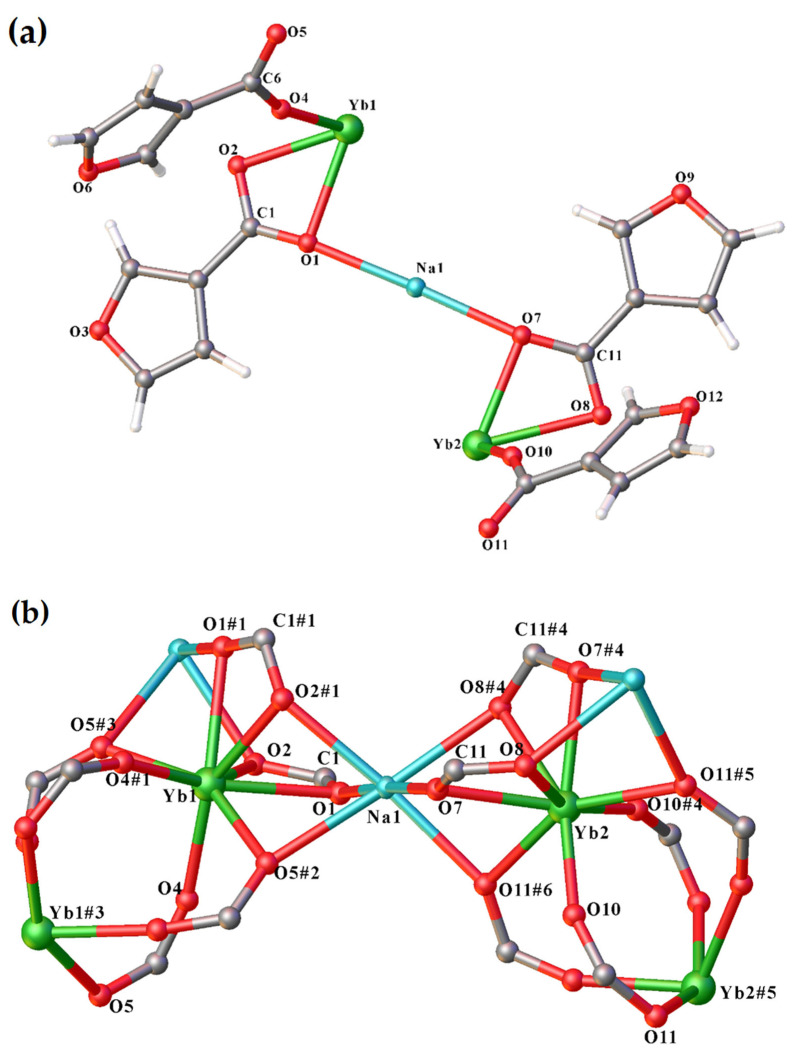

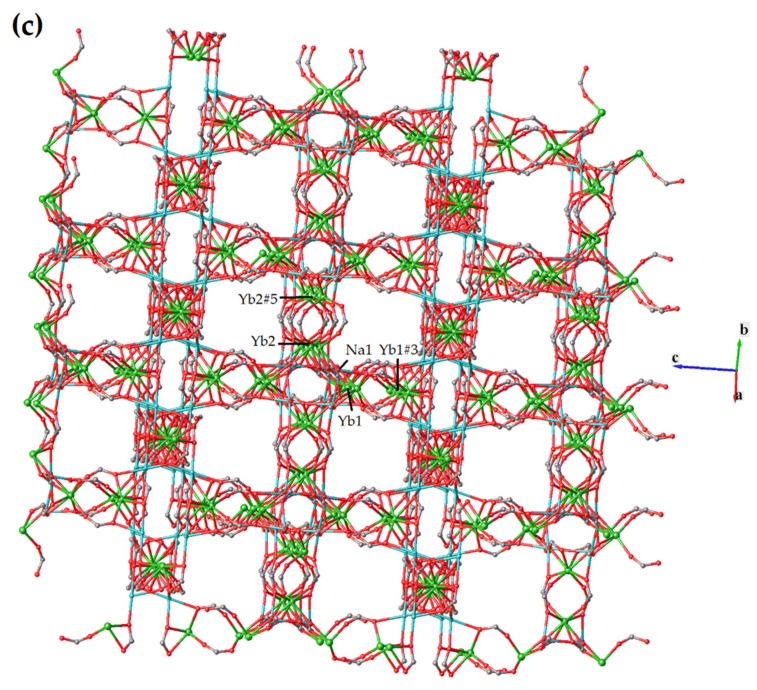
(**a**) Asymmetric unit of [NaYb(3fur)_4_]_n_ (**2Yb**) (representative of **2RE** = 2Ho, 2Y, 2Er, 2Lu) (**b**) The immediate coordination environment around Yb1, Yb2 and Na1 metals atoms in **2Yb** (furan rings are omitted for clarity); Selected bond lengths (Å) and angles (°) (Data for all **2RE** in Supplementary information; Tables S5 and S6). **2Yb** Yb1-O1 2.360(3), Yb1-O2 2.432(3), Yb1-O4 2.208(3), Yb1-O5#2 2.321(3), Yb2-O7 2.317(3), Yb2-O8 2.495(3), Yb2-O10 2.180(3), Yb2-O11#6 2.349(3), Na1-O1 2.317(3), Na1-O2#1 2.338(4), Na1-O5#2 2.428(4), Na1-O7 2.323(4), Na1-O8#4 2.369(4), Na1-O11#6 2.367(4), C1-Yb1-C1#1 85.81(19), C11-Yb2-C11#4 82.54(18), Yb1-Na1-Yb2 175.26(7); (**c**) The 3D framework of **2Yb**; Symmetry code: ^#1^ 1-X,1-Y,+Z; ^#2^ +Y,1-X,1-Z; ^#3^ 1-Y,+X,1-Z; ^#4^ 3/2-Y,3/2-X,3/2-Z; ^#5^ -1/2+Y,1/2+X,3/2-Z; ^#6^ 1-X,2-Y,+Z.

Single crystals of X-ray crystallography quality were obtained for all five Ho, Y, Er, Yb and Lu analogues. The isostructural series of complexes forms a 3D polymeric network and crystallizes in the tetragonal *P*-4*n*2 space group. All RE^3+^ are eight coordinated with two chelating ligands and four bridging ligands in distorted square antiprismatic geometry, Na1 is six-coordinate with six bridging ligands, and the donor atoms have an octahedral arrangement. Specific refinement parameters for individual structures are given in the Supplementary Material. A representative structure [NaYb(3fur)_4_]_n_ of the Type **2RE** can be found in Figure 5. The Yb1 and Yb2 atoms are bridged by the O1-Na1-O7 moiety present in the asymmetric unit of the crystal structure (Figure 5a). The Yb1 metal centre is bonded by chelating carboxylate oxygen atoms O1,2 and O1#1,2#1 of two µ_3_-1κ(O):2κ(O,O′):3κ(O′) ligands. There are also four carboxylate ligands bridging through oxygen atoms O4, 4#1, 5#2, 5#3 of µ_3_- 1κ(O):2κ(O’):3κ(O’) carboxylate groups. The ligand binding modes contained within the coordination sphere of the Yb2 metal atom are similar to those of the above-mentioned Yb1 metal atom, but Yb-O bond distances are different in each case (Table S5). Six coordinated Na1 is ligated through O1, 2#1, 7, 8#4 of four µ_3_-1κ(O):2κ(O,O′):3κ(O′) coordinated modes and the other two sites are occupied by oxygen atoms O5#2 and O11#6 of two µ_3_-1κ(O):2κ(O′):3κ(O′) carboxylate ligands. Yb1 and Na1, and Yb2 and Na1 are bridged by oxygen atoms O1, 2#1, O5#2 and O 7, 8#4, 11#6, respectively (Figure 5b).

The RE-O and Na-O bond lengths are listed in Table S5. The average Yb1-O, Yb2-O and Na1-O bond lengths are 2.330 Å, 2.335 Å and 2.358 Å, correspondingly. The longest RE-O bond length observed in the structure is between Yb2-O8 and Yb2-O8#4 at 2.495(3) Å with Yb2-O10 and Yb2-O10#4 having the shortest RE-O bond distance of 2.180(3) Å. The longest Yb-O bonds are from the oxygen atoms O8 and O8#4 (both chelating and bridging), while the shortest bond lengths are from bridging oxygen atoms O10 and O10#4. The chelating µ_3_-1κ(O):2κ(O,O′):3κ(O′) carboxylate ligands form *cisoid* angles of 85.81(19)° (C1-Yb1-C1#1) and 82.54(18)° (C11-Yb2-C11#4). The metal-metal distance between Yb1….Na1 and Yb2…Na1 are 3.3826(18) and 3.3720(18), respectively, with Yb1-Na1-Yb2 at an angle of 175.26(7)°. The effect of lanthanoid contraction [45] is reflected across the series with the reduction of average RE-O bond distances in 1D chains (Table S5).

Yb1 and Yb1#3 metals are connected by four µ_3_-1κ(O):2κ(O′):3κ( O′) ligands binding through O4-C6-O5, O5#2-C6#2-O4#2, O4#1-C6#1-O5#1 and O5#3-C6#3-O4#3 with all having same O-C-O bond angle of 123.6(4)°, and Yb1…Yb1#3 distance of 4.0237(9) Å. In a similar manner, Yb2 is connected with Yb2#5 by four carboxylates with all of them having an O-C-O bond angle of 123.4(4)°. Yb2 atoms are linked through these bridging oxygens to form dimeric units with a Yb2…Yb2 separation of 4.0006(4) Å and these dimers propagate to form 1D channels. However, the successive Yb2 chains are perpendicular to each other, thus constructing two types of chains. As mentioned above, symmetric bridging between adjacent Yb1^(III)^ atoms leads to the formation of dimeric Yb1 channels that crosslink the neighbouring Yb2 channels running along the perpendicular direction, into a two-dimensional grid. Finally, sodium metal atoms connect all dimers into a 3D coordination polymer, namely [NaYb(3fur)_4_]_n_. (Figure 5c).

### 2.3. Corrosion Inhibition

#### 2.3.1. Immersion Tests

Immersion studies were conducted over seven days in 0.01 M NaCl control solution and in the presence of inhibition systems to access the longer-term performance of these inhibitors for mild steel. The testing was conducted for the compounds at 800 ppm as the solubility limit for [Y(3fur)_3_(H_2_O)_2_]_n_ in 0.01 M sodium chloride solution is close to 800 ppm. However, [Ce(3fur)_3_(H_2_O)_2_]_n_ and [Pr(3fur)_3_(H_2_O)_2_]_n_ compounds had maximum solubilities as high as 3000 ppm.

Table 2 presents a summary of the weight loss measurements after 168 h of immersion in specific inhibitor solutions. The corrosion rate (R) of each inhibitor compound was evaluated and then percentage corrosion inhibition (η) was calculated using Equation (1).

(1) $$\eta =\frac{\;R\left(Control\right)-R\;\left(inhibitor\right)}{R\left(Control\right)}\;\times \;100$$

The corrosion rates of the samples placed in the inhibitor solutions were found to be significantly lower than that of control coupons. As shown in Table 2, [Y(3fur)_3_(H_2_O)_2_]_n_ compound has the highest inhibitor efficiency of 90%. Furthermore, the visual inspection of the surface immersed in [Y(3fur)_3_(H_2_O)_2_]_n_ containing solutions also suggests a minimum corrosive attack on the steel surface with relatively less surface roughness (Figure S6). As a group, Type **1RE** [RE(3fur)_3_(H_2_O)_2_]_n_ complexes showed better inhibition performance than the Type **2RE** bimetallic [NaRE(3fur)_4_]_n_ species. This was evident from the better level of protection achieved by Type **1RE** monometallic Y^III^ and Ho^III^ inhibited solutions compared to the Type **2RE** Na-Y and Na-Ho bimetallic compounds. This may be a consequence of the presence of coordinated sodium in the compounds in the latter instance. The influence of sodium on inhibition efficiency can be seen by comparing the immersion test results of sodium 3-furoate (Na(3fur)) with the rare earth carboxylate complexes in Table 2. It has also been shown in previous literature that the corrosion rates of sodium carboxylates on steel are generally higher than that of rare earth analogues [46]. However, the **2Yb** complex is a positive outlier since it gave the second-best performance with an η of 88%.

When considering lighter rare earths (La-Nd) the efficiency decreased from La-Nd. The level of protection by the heavy rare earths correlates with the decreasing atomic radius of the rare earth metals since the inhibitive effect increased from Gd-Y among the [RE(3fur)_3_(H_2_O)_2_]_n_ class. A similar trend was observed to some extent with [NaRE(3fur)_4_]_n_ complexes with improved protection from Ho to Yb, but Lu is a negative outlier. The low solubility of most of the complexes has hampered attempts at concentration studies. The performance was better than of the analogous rare earth 3-thiophenecarboxylates [31], suggesting that an oxygen substituent may be more beneficial than sulfur, but the 3fur complexes were tested at somewhat higher concentration owing to greater solubility.

#### 2.3.2. Potentiodynamic Polarisation

The results of the potentiodynamic polarisation (PP) experiments are shown in Table 3. [Y(3fur)_3_(H_2_O)_2_]_n_ **1Y** showed the lowest i_corr_, at 0.56 µA/cm^2^, and **1Y** > **2Yb** > **1La** in effectiveness. After 24 h immersion, with the compounds present in the solution there is a shift in corrosion potential (E_corr_) values towards more positive direction compared to the control solution, indicative of anodic inhibition dominating the reduction in i_corr_.

**Figure 6:**
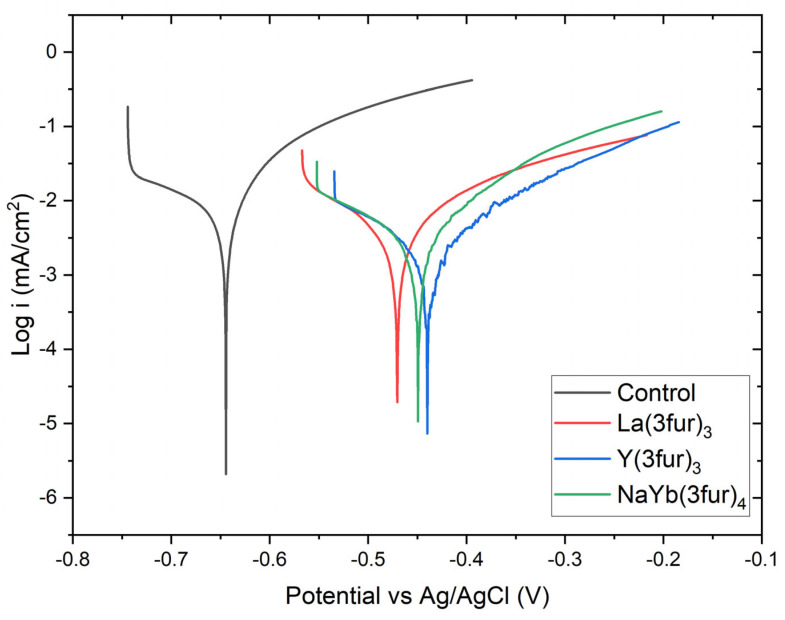
Representative potentiodynamic polarisation scans after 24 h for 0.01 M NaCl control, and solutions with 0.01 M NaCl and 800ppm of [Y(3fur)_3_(H_2_O)_2_]_n_ (**1Y**), [La(3fur)_3_(H_2_O)_2_]_n_ (**1La**) and [NaYb(3fur)_4_]_n_ (**2Yb**).

Representative PP scans in Figure 6 show the marked reduction in the anodic branch with the addition of inhibitors, and some reduction in the cathodic kinetics. According to the previous literature [46], this is a typical behaviour of these types of compounds. However, the efficiency of the [Y(3fur)_3_(H_2_O)_2_]_n_ was somewhat inferior to that of yttrium 3-(4′-methylbenzoyl)propanoate [6], which though is limited by low solubility. For this reason, yttrium 3-furoate may potentially be more useful.

## 3. Materials and Methods

### 3.1. General Consideration

All reagents and solvents that were used are of standard commercial grade and used without further purification. IR spectra were collected using a Nicolet™ iS™ 5 FTIR Spectrometer in the range of 4000–500 cm^−1^. Elemental analyses were performed by the Elemental Analysis Service Team, Science Centre, London Metropolitan University, England. Metal analysis was conducted by complexometric titration with 0.01 M EDTA using Xylenol Orange indicator and hexamethylenetetramine buffer. Thermogravimetric analysis (TGA) was conducted on a TA instrument SDT 650 using standard 90 µL alumina metal pans under an N_2_ atmosphere (50 ml min^−1^) from room temperature up to 750 °C (with a ramp of 10 °C min^−1^). Melting points were determined in glass capillaries and are reported uncalibrated. Powder XRD measurements were obtained at room temperature using a Bruker D2 PHASER diffractometer in the range of 2–60° with a 0.2° divergence slit and at 0.02° increments. X-ray powder simulations were generated using the Mercury program provided by Cambridge Crystallographic Data Centre [47], from the obtained single-crystal X-ray diffraction data. LCMS was collected on an Agilent 6100 Series Single Quad LC/MS coupled with an Agilent 1200 Series HPLC.

### 3.2. X-ray Crystallography

Single crystals were mounted on loops using viscous hydrocarbon oil. Data were collected on the MX1 beamline at the Australian Synchrotron. The data integration was completed using Blue-ice [48] and XDS [49] software programs. Structures were solved by SHELXT and refined by full-umatrix least-squares methods against F2 using SHELX2018 [50], utilizing the Olex2 [51] graphical user interface. All hydrogen atoms were placed in calculated positions using the riding model. The graphical representations were generated using bitmap images GUI of Olex2 [51]. Crystal data and refinement details are given in Table S1.

### 3.3. Synthesis of [RE(3fur)_3_(H_2_O)_2_]_n_ and [NaRE(3fur)_4_]_n_ Complexes

General Synthetic Method: 3-furoic acid was dissolved in 95% ethanol (15 mL) and deprotonated with an equimolar amount of aqueous sodium hydroxide. The solution was stirred for 1 h and the pH was adjusted to 7–8. Treatment of the sodium 3-furoate with a hydrated rare earth chloride or nitrate (mole ratio 3:1) in water at pH 5 gave rare earth3-furoate complexes (**1RE** = 1La, 1Ce, 1Pr, 1Nd, 1Gd, 1Dy, 1Ho, 1Y and **2RE** = 2Er, 2Yb, 2Lu). Under similar reaction conditions, the metathesis reaction of the preformed ligand and the corresponding rare earth salt with 4:1 stoichiometry resulted in the Na-RE bimetallic 3-furoate complexes **2RE** = 2Ho, 2Y, 2Er, 2Yb and 2Lu, but not for La-Dy, which gave 1La-1Dy**.** The resultant precipitates were filtered off and air-dried for 2–3 days. Crystals were obtained upon slow evaporation of the mother liquor at room temperature.

**1La**: [La(3fur)_3_(H_2_O)_2_]_n_ white powder. Yield: 65%. m.p. 245 °C (dec). Elemental analysis for C_15_H_13_LaO_11_ (MW: 508.16 gmol^−1^): Calculated (%) C 35.45; H 2.58; La 27.33. Found (%) C 35.57; H 2.42; La 27.50. IR (cm^−1^): 3421 w, 3132 w, 1575 s, 1507 s, 1437 s, 1364 s, 1235 m, 1219 m, 1209 m, 1151 m, 1068 m, 1003 m, 971 m, 875 s, 851 w, 784 s, 741 s, 731 m, 598 s, 550 s, 531 s, 435 s. TGA weight loss (76–131 °C); 7.0% (Calc. for loss of 2 × H_2_O = 7.1%); weight loss (328–420 °C); 31.0% (Calc. for the formation of La_2_(CO_3_)_3_ = 47.9%).

**1Ce:** [Ce(3fur)_3_(H_2_O)_2_]_n_ white powder. Yield: 76%. m.p.260 °C (dec). Elemental analysis for C_15_H_13_CeO_11_ (MW: 509.37 gmol^−1^): Calculated (%) C 35.37; H 2.57; Ce 27.51. Found (%) C 35.52; H 2.40; Ce 27.64. IR (cm^−1^):3439 w, 3134 w, 1574 s, 1505 s, 1437 s, 1364 s, 1235 m, 1220 m, 1209 m, 1151 m, 1068 m, 1003 m, 972 m, 874 s, 851 w, 783 s, 741 s, 731 m, 598 s, 550 s, 531 s, 434 s. TGA weight loss (71–120 °C); 6.9% (Calc. for loss of 2 × H_2_O = 7.1%); weight loss (364–475 °C); 31.9% (Calc. for the formation of Ce_2_(CO_3_)_3_ = 47.8%).

**1Pr:** [Pr(3fur)_3_(H_2_O)_2_]_n_ pale green powder. Yield: 80%. m.p.240 °C (dec). Elemental analysis for C_15_H_13_O_11_Pr (MW: 510.16 gmol^−1^): Calculated (%) C 35.31; H 2.57; Pr 27.62. Found (%) C 35.39; H 2.39; Pr 27.42. IR (cm^−1^): 3442 w, 3133 w, 1573 s, 1525 w, 1504 s, 1434 s, 1364 s, 1234 m, 1220 m, 1209 m, 1150 m, 1069 m, 1003 m, 972 m, 875 s, 852 w, 786 s, 741 s, 730 m, 598 s, 552 s, 533 s, 438 s. TGA weight loss (70–154 °C); 6.9% (Calc. for loss of 2 × H_2_O = 7.1%); weight loss (368–492 °C); 31.7% (Calc. for the formation of Pr_2_(CO_3_)_3_ = 47.7%).

**1Nd:** [Nd(3fur)_3_(H_2_O)_2_]_n_ purple powder. Yield: 70%, m.p.260 °C (dec). Elemental analysis for C_15_H_13_NdO_11_ (MW: 513.49 gmol^−1^): Calculated (%) C 35.08; H 2.55; Nd 28.09. Found (%) C 35.35; H 2.33; Nd 27.94. IR (cm^−1^): 3431 w, 3134 w, 1573 s, 1527 w, 1505 s, 1439 s, 1364 s, 1234 m, 1221 m, 1209 m, 1151 m, 1069 m, 1003 m, 973 m, 875 s, 852 w, 783 s, 741 s, 730 m, 598 s, 553 s, 534 s, 439 s. TGA weight loss (76–123 °C); 6.9% (Calc. for loss of 2 × H_2_O = 7.0%); weight loss (365–495 °C); 32.4% (Calc. for the formation of Nd_2_(CO_3_)_3_ = 47.4%).

**1Gd:** [Gd(3fur)_3_(H_2_O)_2_]_n_ white powder. Yield: 60%. m.p.260 °C (dec). Elemental analysis for C_15_H_13_GdO_11_ (MW: 526.50 gmol^−1^): Calculated C 34.22; H 2.49; Gd 29.87. Found (%) C 33.91; H 2.39; Gd 29.62. IR (cm^−1^): 3440 w, 3134 w, 1574 s, 1537 w, 1505 s, 1443 s, 1365 s, 1234 m, 1222 m, 1209 m, 1151 m, 1068 m, 1002 m, 973 m, 875 s, 854 w, 784 s, 742 s, 730 m, 598 s, 556 s, 533 s, 438 s TGA weight loss (75–130°C); 6.9% (Calc. for loss of 2 × H_2_O = 6.8 %); weight loss (374–490 °C); 30.2% (Calc. for the formation of Gd_2_(CO_3_)_3_ = 46.2%).

**1Dy:** [Dy(3fur)_3_(H_2_O)_2_]_n_ white powder. Yield: 79%. m.p.260 °C (dec). Elemental analysis for C_15_H_13_DyO_11_ (MW: 531.75 gmol^−1^): Calculated (%) C 33.88; H 2.46; Dy 30.56. Found (%) C 33.52; H 2.34; Dy 30.70. IR (cm^−1^): 3420 w, 3135 w, 1574 s, 1534 m, 1505 s, 1437 s, 1364 s, 1234 m, 1223 m,1207 m, 1151 s, 1068 m, 1003 m, 974 m, 875 s, 855 w, 783 s, 742 s, 730 m, 598 s, 559 s, 534 s, 442 s. LCMS: (m/z): Calc. for [Dy(3fur)(OH).EtOH]^+^ 338.0, [Dy(3fur)_2_(H_2_O)_2_]^+^ 422.0; found 337.1, 422.1. TGA weight loss (60–124 °C); 6.7% (Calc. for loss of 2 × H_2_O = 6.8%); weight loss (390–482 °C); 30.1% (Calc. for the formation of Dy_2_(CO_3_)_3_ = 45.7%).

**1Ho:** [Ho(3fur)_3_(H_2_O)_2_]_n_ yellow powder. Yield: 61%. m.p.260 °C (dec). Elemental analysis for C_15_H_13_HoO_11_ (MW: 534.18 gmol^−1^): Calculated (%) C 33.73; H 2.45; Ho 30.87. Found (%) C 33.75; H 2.35; Ho 30.84. IR (cm^−1^): 3441 w, 3135 w, 1575 s, 1535 m, 1501 s, 1444 s, 1365 s, 1234 m, 1223 m, 1210 m, 1152 s, 1068 m, 1003 m, 973 m, 874 s, 855 m, 782 s, 742 s, 730 m, 598 s, 560 s, 533 s, 442 s. TGA weight loss 63–110 °C); 6.9% (Calc. for loss of 2 × H_2_O = 6.7%); weight loss (380–491 °C); 31.5% (Calc. for the formation of Ho_2_(CO_3_)_3_ = 45.5%).

**1Y:** [Y(3fur)_3_(H_2_O)_2_]_n_ white powder. Yield: 67%. m.p.230 °C (dec). Elemental analysis for C_15_H_13_O_11_Y (MW: 458.16 gmol^−1^): Calculated (%) C 39.32; H 2.86; Y 19.40. Found (%) C 39.64; H 2.62; Y 19.80. IR (cm^−1^): 3441 w, 3136 w, 1575 s, 1538 m, 1505 s, 1445 s, 1366 s, 1234 m, 1223 m, 1209 m, 1151 s, 1068 m, 1002 m, 974 m, 875 s, 855 m, 784 s, 742 s, 724 m, 598 s, 561 s, 531 s, 441 s. TGA weight loss (55–135 °C); 7.9% (Calc. for loss of 2 × H_2_O = 7.9%); weight loss (372–498 °C); 35.1% (Calc. for the formation of Y_2_(CO_3_)_3_ = 53.1%).

**2Ho:** [HoNa(3fur)_4_]_n_ yellow powder. Yield: 56%. m.p.200 °C (dec). Elemental analysis for C_20_H_12_HoNaO_12_ (MW: 632.22 gmol^−1^): Calculated (%) C 38.00; H 1.91; Ho 26.09. Found (%) C 37.52; H 1.90; Ho 26.11. IR (cm^−1^): 3131 w, 2363 w, 1619 m, 1577 s, 1541 s, 1502 s, 1434 s, 1366 s, 1237 m, 1212 s, 1154 s, 1075 m, 1007 m, 971 m, 872 s, 840 w, 824 s, 815 s, 804 s, 776 s, 737 s, 598 s, 564 w, 539 w, 457 s. LCMS: (m/z): Calc. for Na_2_Ho(3fur)_3_(3furH)(HCO_3_)(H_2_O)]^+^ 735.0, [Ho_2_(3fur)_4_(HCO_3_)(H_2_O)]^+^ 853.0; found 735.2, 853.5. TGA weight loss (403–454 °C); 33.1% (Calc. for the formation of Na_3_Ho(CO_3_)_3_ = 34.6%).

**2Y:** [NaY(3fur)_4_]_n_ white powder. Yield: 51%, m.p.240 °C (dec). Elemental analysis for C_20_H_12_NaO_12_Y (MW: 556.20 gmol^−1^): Calculated (%) C 43.19; H 2.17; Y 15.98. Found (%) C 42.54; H 1.87; Y 15.81. IR (cm^−1^): 3131 w, 2361 w, 1619 m, 1577 s, 1543 s, 1503 s, 1434 s, 1366 s, 1237 m, 1212 s, 1154 s, 1075 m, 1007 m, 971m, 872s, 841 w, 824 s, 816 s, 804 s, 776 s, 737 s, 598 s, 563 w, 539 w, 461 s. TGA weight loss (410–457 °C); 37.1% (Calc. for the formation of Na_3_Y(CO_3_)_3_ = 39.2%).

**2Er:** [NaEr(3fur)_4_]_n_ pink powder. Yield: 59%. m.p.240 °C (dec). Elemental analysis for C_20_H_12_ErNaO_12_ (MW: 634.55 gmol^−1^): Calculated (%) C 37.86; H 1.91; Er 26.36. Found (%) C 37.78; H 1.83; Er 26.54. IR (cm^−1^): 3136 w, 2361 w, 1621 m, 1577 s, 1542 s, 1502 s, 1433 s, 1366 s, 1237 m, 1213 s, 1154 s, 1075 m, 1007 m, 971 m, 873 s, 840 w, 824 s, 816 s, 804 s, 776 s, 737 s, 598 s, 566 w, 539 w, 457 s; TGA weight loss (400–453 °C); 32.2% (Calc. for the formation of Na_3_Er(CO_3_)_3_ = 34.4%).

**2Yb:** [NaYb(3fur)_4_]_n_ white powder. Yield: 60%. m.p.240 °C (dec). Elemental analysis for C_20_H_12_YbNaO_12_ (MW: 640.33 gmol^−1^): Calculated (%) C 37.51; H 1.89; Yb 27.02. Found (%) C 36.35; H 1.82; Yb 27.03. IR (cm^−1^): 3132 w, 2360 w, 1624 m, 1578 s, 1545 s, 1502 s, 1434 s, 1366 s, 1237 m, 1213 s, 1154 s, 1075 m, 1007 m, 971 m, 873 s, 840 w, 825 s, 818 s, 804 s, 776 s, 738 s, 598 s, 567 w, 540 w, 457 s. TGA weight loss (400–452 °C); 31.3% (Calc. for the formation of Na_3_Yb(CO_3_)_3_ = 34.1%).

**2Lu:** [NaLu(3fur)_4_]_n_ white powder. Yield: 51%. m.p.250 °C (dec). Elemental analysis calculated for C_20_H_12_LuNaO_12_ (MW: 642.26 gmol^−1^) Calculated (%) C 37.40; H 1.88; Lu 27.24. Found (%) C 36.75; H 1.82; Lu 27.31. IR (cm^−1^): 3132 w, 2360 w, 1626 m, 1578 s, 1547 s, 1503 s, 1435 s, 1366 s, 1238 m, 1213 s, 1154 s, 1075 m, 1007 m, 971 m, 873 s, 840 w, 826 s, 818 s, 804 s, 776 s, 738 s, 598 s, 568 w, 540 w, 459 s. LCMS: (*m/z*): Calc. for [Na_2_(CO_3_)Lu(3fur)_4_(H_2_O)_2_]^−^ 761.0; found 761.0. TGA weight loss (397–450 °C); 32.2% (Calc. for the formation of Na_3_Lu(CO_3_)_3_ = 33.9%).

### 3.4. Corrosion Testing

To evaluate the general corrosion and inhibition behaviour of synthesized compounds, corrosion immersion experiments were conducted according to the standard method ASTM G31-72 [52]. Mild steel alloy AS 1020 coupons used in the weight loss tests were cut to approximately 20 × 20 × 1.5 mm and abraded progressively with sanding sheets of 80, 120, 240, 360, 800, 1200 and 2000 grits. The specimens were rinsed with distilled water followed by ethanol and dried under flowing N_2_ gas. The coupons were used immediately after polishing and washing to do a series of immersion tests, up to 168 h (7 days). The sample and the control coupons were suspended in beakers containing 0.01 M NaCl solutions with and without 800 ppm (1.25–1.75 mM) of the inhibitor compounds. In each setup, replicates were done by fully immersing the coupons at mid-depth with the use of Teflon strings. Upon the completion of the test, firstly, the corrosion product that clung to the substrate was removed by mild sonication in clean distilled water followed by using the finest sanding papers with minimum force to avoid the removal of sound material. Experiments were done in duplicate. Lastly, the coupons were washed with ethanol and dried with N_2_ gas.

The compounds containing the REs Y, Yb and La were used to conduct Potentiodynamic Polarisation (PP) experiments, as they showed the best performance in weight loss experiments. The tests were performed using a Bio-Logic VMP3 multi-channel potentiostat with solutions containing the same concentrations as those used in the weight loss experiments. A three-electrode cell with a titanium mesh counter electrode, a Ag/AgCl reference electrode and an AS1020 mild steel rod as the working electrode surface was used. The working electrode consisted of a 10mm diameter steel rod steel rod in epoxy, polished to a 1200 grit finish. A test solution volume of 100mL was used, with the solution open to air. Open Circuit Voltage (OCV) was monitored for 24 h following which the PP scan was conducted over a scan range of 150 mV below to 250 mV above OCV at 0.167 mV/s. A Tafel extrapolation was used to determine the corrosion current densities (i_corr_) and potentials (E_corr_) from the PP curves using EC Lab software V11.27.

## 4. Conclusions

A series of novel rare earth 3-furoate complexes was synthesized and characterized. The 13 structures identified in this study could be separated into two groups based on their molecular composition as below.

- **Type 1**-Monometallic 2D polymeric network of [RE(3fur)_3_(H_2_O)_2_]_n_
    (**1RE** = 1La, 1Ce, 1Pr, 1Nd, 1Gd, 1Dy, 1Ho, 1Y)
- **Type 2**-Bimetallic 3D polymeric network of [NaRE(3fur)_4_]_n_
    (**2RE** = 2Ho, 2Y, 2Er, 2Yb, 2Lu)

The ability to obtain two different structural motifs from HoCl_3_ and YCl_3_ at 1:3 and 1:4 (rare earth: ligand) mole ratios indicates that the stoichiometry used in the metathesis reaction affects the final complex formation. The lanthanoid contraction was evident with a reduction of the average RE-O bond distance across each series.

The immersion studies showed that at 800 ppm inhibitor concentrations for mild steel in 0.01 M NaCl solutions, [Y(3fur)_3_(H_2_O)_2_]_n_ (**2Y**) was the best performing inhibitor with [NaYb(3fur)_4_]_n_ (**2Yb**) and [La(3fur)_3_(H_2_O)_2_]_n_ (**1La**) exhibiting comparable inhibition performance. Potentiodynamic polarisation confirmed the superior performance of [Y(3fur)_3_(H_2_O)_2_]_n_ (**1Y**). The compounds act predominantly as anodic inhibitors. A comparison of Y complex data suggests that the introduction of a furoate moiety into a carboxylate is more effective than the thiophene group but not as effective as a 3-(4′-methylbenzoyl) group [6,31]. Application of the rare earth 3-furoates as inhibitors would involve dissolution of the solid complexes in the water of cooling towers or radiators, etc., to give a very dilute solution. In protective coating, a suspension of the complexes in paint would be used, but the effectiveness in this role has still to be investigated.

## Figures and Tables

**Table 1:** Selected infrared bands (cm^−1^) of the RE^III^ 3-furoate complexes.

Compound	ν(OH)_water_	ν_as_(CO_2_^−^)	ν_s_(CO_2_^−^)	Δν = (ν_as_−ν_s_)avg	δ(CO_2_)
[Nd(3fur)_3_(H_2_O)_2_]_n_ (**1Nd**)	3431	1505	1365	140	598
[NaYb(3fur)_4_]_n_ (**2Yb**)	-	1502	1366	136	598

**Table 2:** Observed weight loss, corrosion rates (g/m^2^h) and percentage inhibition (η) for mild steel coupons immersed in specific solutions at 800 ppm concentrations in 0.01 M NaCl for seven days.

Solution	Concentration		Solubility in 0.01 M NaCl (ppm)	Avg Weight Loss (mg)	Corrosion Rate (g/m^2^h)	Inhibition, η (%)
	ppm	mM				
Control-NaCl	580	10	-	19.6	1406.1	-
[La(3fur)_3_(H_2_O)_2_]_n_ **1La**	800	1.57	2000	2.6	185.2	87
[Ce(3fur)_3_(H_2_O)_2_]_n_ **1Ce**	800	1.57	3000	3.7	298.6	79
[Pr(3fur)_3_(H_2_O)_2_]_n_ **1Pr**	800	1.57	3000	6.1	380.7	73
[Nd(3fur)_3_(H_2_O)_2_]_n_ **1Nd**	800	1.56	2500	5.1	365.9	74
[Gd(3fur)_3_(H_2_O)_2_]_n_ **1Gd**	800	1.57	1000	6.2	403.9	71
[Dy(3fur)_3_(H_2_O)_2_]_n_ **1Dy**	800	1.52	830	3.0	229.6	84
[Ho(3fur)_3_(H_2_O)_2_]_n_ **1Ho**	800	1.50	920	2.9	210.9	85
[Y(3fur)_3_(H_2_O)_2_]_n_ **1Y**	800	1.75	800	1.9	140.6	90
[NaHo(3fur)_4_]_n_ **2Ho**	800	1.27	1800	6.0	431.7	70
[NaY(3fur)_4_]_n_ **2Y**	800	1.44	1900	4.9	355.1	75
[NaEr(3fur)_4_]_n_ **2Er**	800	1.26	1800	5.1	365.9	74
[NaYb(3fur)_4_]_n_ **2Yb**	800	1.25	1700	2.4	168.7	88
[NaLu(3fur)_4_]_n_ **2Lu**	800	1.25	1500	7.7	480.6	66
[Na(3fur)]	800	5.97	>7000	16.51	993.6	29

**Table 3:** Summary of corrosion parameters obtained from potentiodynamic polarisation tests in control and inhibited solutions after 24 h immersion.

Solution	i_corr_ (µA/cm^2^)	Std. Dev.	E_corr_ (mV)	Std. Dev.	Percentage Inhibition
Control-NaCl	2.88	0.11	−638	4	
[La(3fur)_3_(H_2_O)_2_]_n_ **1La**	1.09	0.09	−473	5	62
[Y(3fur)_3_(H_2_O)_2_]_n_ **1Y**	0.56	0.08	−445	4	80
[NaYb(3fur)_4_]_n_ **2Yb**	0.89	0.07	−468	29	69

## Data Availability

Crystal data can be obtained free of charge from The Cambridge Crystallographic Data Centre (CCDC 2218918-2218925 for complexes **1RE**, and 2218926-2218930 for complexes **2RE**).

## Supplementary Materials

The following supporting information can be downloaded at: https://www.mdpi.com/article/10.3390/molecules27248836/s1, Figure S1: IR spectra of [RE(3fur)_3_(H_2_O)_2_]_n_ **1RE** series; Figure S2: IR spectra of [NaRE(3fur)_4_]_n_ **2RE** series; Figure S3: TGA plots of the [RE(3fur)_3_(H_2_O)_2_]_n_ **1RE** series; Figure S4: TGA plots of the [NaRE(3fur)_4_]_n_ **2RE** series; Figure S5: IR spectra of Ce_2_(CO_3_)_3_ (Left) from thermal decomposition of **1Ce**, reported Dy_2_(CO_3_)_3_⋅4H_2_O (middle) and Na_3_Yb(CO_3_)_3_ (right) from thermal decomposition of **2Yb**; Figure S6: Mild steel coupons immersed in control and the three best inhibited solutions for 168 h (Left—Trial 1; right—Trial 2.); Table S1: Crystal data and structural refinement for rare earth 3-furoate complexes; Table S2: Selected bond lengths and RE…RE distances (Å) for the isostructural [RE(3fur)_3_(H_2_O)_2_]_n_ **1RE** series; Table S3: Selected bond angles (°) for the isostructural [RE(3fur)_3_(H_2_O)_2_]_n_ **1RE** series; Table S4: Hydrogen bonds for [RE(3fur)_3_(H_2_O)_2_]_n_ (**1Nd**) [d/Å and </°]; Table S5: Selected bond lengths and Na…RE distances (Å) for the isostructural [NaRE(3fur)_4_]_n_ **2RE** series; Table S6: Selected bond angles (°) for the isostructural [NaRE(3fur)_4_]_n_ **2RE** series.

## References

[1] Odio O.B., Chinwuko C.E., Chukwuneke L.J., Sinebe E.J. (2014). Investigation of The Effect of Corrosion on Mild Steel in Five Different Environments. Int. J. Sci. Technol. Res..

[2] Garcia S.J., Mol J.M.C., de Wit J.H.W., Muster T.H., Hughes A.E., Miller T., Markley T., Mardel J., Terryn H., Fedrizzi L., Fürbeth W., Montemor F. (2011). Advances in the Selection and Use of Rare-Earth-Based Inhibitors for Self-Healing Organic Coatings. Self-Healing Properties of New Surface Treatments.

[3] Hossain N., Chowdhury M.A., Kchaou M. (2020). An overview of green corrosion inhibitors for sustainable and environment friendly industrial development. J. Adhes. Sci. Technol..

[4] Mottram E., Hamilton S., Moon J.S., Wang J., Bousrez G., Somers A.E., Deacon G.B., Junk P.C. (2020). Synthesis, structure, and corrosion inhibiting properties of phenylacetato-rare earth (III) complexes. J. Coord. Chem..

[5] Bowman E., Thompson N., Gl D., Moghissi O., Gould M., Payer J. (2016). International Measures of Prevention, Application, and Economics of Corrosion Technologies Study.

[6] Somers A.E., Hinton B.R., de Bruin-Dickason C., Deacon G.B., Junk P.C., Forsyth M. (2018). New, environmentally friendly, rare earth carboxylate corrosion inhibitors for mild steel. Corros. Sci..

[7] Forsyth M., Wilson K., Behrsing T., Forsyth C., Deacon G.B., Phanasgoankar A. (2002). Effectiveness of Rare-Earth Metal Compounds as Corrosion Inhibitors for Steel. Corrosion.

[8] Chong A.L., Mardel J.I., MacFarlane D.R., Forsyth M., Somers A.E. (2015). Synergistic Corrosion Inhibition of Mild Steel in Aqueous Chloride Solutions by an Imidazolinium Carboxylate Salt. ACS Sustain. Chem. Eng..

[9] Ghorbani M., Soto Puelles J., Forsyth M., Catubig R.A., Ackland L., Machuca L., Terryn H., Somers A.E. (2020). Corrosion Inhibition of Mild Steel by Cetrimonium Trans-4-Hydroxy Cinnamate: Entrapment and Delivery of the Anion Inhibitor through Speciation and Micellar Formation. J. Phys. Chem. Lett..

[10] Forsyth M., Seter M., Hinton B., Deacon G., Junk P. (2011). New “Green” Corrosion Inhibitors Based on Rare Earth Compounds. Aust. J. Chem..

[11] Peng Y., Hughes A.E., Deacon G.B., Junk P.C., Hinton B.R., Forsyth M., Mardel J.I., Somers A.E. (2018). A study of rare-earth 3-(4-methylbenzoyl)-propanoate compounds as corrosion inhibitors for AS1020 mild steel in NaCl solutions. Corros. Sci..

[12] Hinton B., Gschneidner K.A., Eyring L. (1995). Corrosion Prevention and Control. Handbook on the Physics and Chemistry of Rare Earths.

[13] Hinton B.R.W. (1992). Corrosion Inhibition with Rare Earth Metal Salts. J. Alloys Compd..

[14] Mendez J.A.C., Vong Y.M., Bueno J.D.J.P. (2022). Cerium and Other Rare Earth Salts as Corrosion Inhibitors—A Review. Prot. Met. Phys. Chem. Surf..

[15] Somers A.E., Deacon G.B., Hinton B.R.W., Macfarlane D.R., Junk P.C., Tan M.Y.J., Forsyth M. (2016). Recent Developments in Environment-Friendly Corrosion Inhibitors for Mild Steel. J. Indian Inst. Sci..

[16] Somers A.E., Peng Y., Chong A.L., Forsyth M., MacFarlane D.R., Deacon G.B., Hughes A.E., Hinton B.R.W., Mardel J.I., Junk P.C. (2020). Advances in the Development of Rare Earth Metal and Carboxylate Compounds as Corrosion Inhibitors for Steel. Corros. Eng. Sci. Technol..

[17] Forsyth M., Hinton B. (2014). Rare Earth-Based Corrosion Inhibitors.

[18] Sinko J. (2001). Challenges of chromate inhibitor pigments replacement in organic coatings. Prog. Org. Coat..

[19] Winkler D.A., Breedon M., Hughes A.E., Burden F.R., Barnard A.S., Harvey T.G., Cole I. (2014). Towards Chromate-Free Corrosion Inhibitors: Structure-Property Models for Organic Alternatives. Green Chem..

[20] Daoud D., Douadi T., Hamani H., Chafaa S., Al-Noaimi M. (2015). Corrosion Inhibition of Mild Steel by Two New S-Heterocyclic Compounds in 1 M HCl: Experimental and Computational Study. Corros. Sci..

[21] Verma C., Ebenso E.E., Quraishi M.A., Saleh H.E.-D.M., Koller M. (2018). Ionic Liquids as Green Corrosion Inhibitors for Industrial Metals and Alloys. Green Chemistry.

[22] Pham T.H., Lee W.-H., Son G.-H., Tran T.T., Kim J.-G. (2022). Synthesis and Corrosion Inhibition Potential of Cerium/Tetraethylenepentamine Dithiocarbamate Complex on AA2024-T3 in 3.5% NaCl. Materials.

[23] Vaidyanathan H., Hackerman N. (1971). Effect of furan derivatives on the anodic dissolution of Fe. Corros. Sci..

[24] Khaled K.F. (2010). Understanding Corrosion Inhibition of Mild Steel in Acid Medium by Some Furan Derivatives: A Comprehensive Overview. J. Electrochem. Soc..

[25] Ouchi A., Suzuki Y., Ohki Y., Koizumi Y. (1988). Structure of Rare Earth Carboxylates in Dimeric and Polymeric Forms. Coord. Chem. Rev..

[26] Mehrotra R.C., Bohra R. (1983). Metal Carboxylates.

[27] Janicki R., Mondry A., Starynowicz P. (2017). Carboxylates of Rare Earth Elements. Coord. Chem. Rev..

[28] Behrsing T., Deacon G.B., Junk P.C., Forsyth M., Hinton B. (2014). The Chemistry of Rare Earth Metals, Compounds, and Corrosion Inhibitors. Rare Earth-Based Corrosion Inhibitors.

[29] Markley T., Blin F., Forsyth M., Hinton B., Forsyth M., Hinton B. (2014). Multifunctional Rare Earth Organic Corrosion Inhibitors. Rare Earth-Based Corrosion Inhibitors.

[30] Blin F., Leary S.G., Deacon G.B., Junk P.C., Forsyth M. (2006). The Nature of the Surface Film on Steel Treated with Cerium and Lanthanum Cinnamate Based Corrosion Inhibitors. Corros. Sci..

[31] Vithana V.P., Guo Z., Deacon G.B., Somers A.E., Junk P.C. (2022). Synthesis, Structure, and Corrosion Inhibiting Properties of RE^III^ 3-Thiophenecarboxylate Complexes. N. J. Chem..

[32] Lajunen L.H., Choppin G.R. (1989). Analytical Chemistry of the Lanthanides Part 1. Atomic Absorption and Plasma Atomic Emission Spectroscopic Methods. Rev. Anal. Chem..

[33] Sitzmann H., Dezember T., Schmitt O., Weber F., Wolmershäuser G., Ruck M. (2000). Metallocenes of Samarium, Europium, and Ytterbium with the Especially Bulky Cyclopentadienyl Ligands C5H(CHMe2)4, C5H2(CMe3)3, and C5(CHMe2)5. Z. Anorg. Allg. Chem..

[34] Daniels D.P., Deacon G.B., Harakat D., Jaroschik F., Junk P.C. (2012). Synthesis and Characterisation of Alkaline Earth (Diphenylphosphano)Metallocene Complexes and Heterobimetallic Alkaline Metal/Platinum (II) Complexes [Ae(Thf)_x_(η^5^-C_5_H_4_PPh_2_)_2_Pt(Me)_2_] (Ae = Ca, Sr, Ba). Dalton Trans..

[35] Moxey G.J., Blake A.J., Lewis W., Kays D.L. (2015). Alkaline Earth Complexes of a Sterically Demanding Guanidinate Ligand. Eur. J. Inorg. Chem..

[36] Kelly R.P., Bell T.D.M., Cox R.P., Daniels D.P., Deacon G.B., Jaroschik F., Junk P.C., le Goff X.F., Lemercier G., Martinez A. (2015). Divalent Tetra- and Penta-Phenylcyclopentadienyl Europium and Samarium Sandwich and Half-Sandwich Complexes: Synthesis, Characterization, and Remarkable Luminescence Properties. Organometallics.

[37] Hirneise L., Maichle-Mössmer C., Anwander R. (2021). Pentamethylcyclopentadienyl Complexes of Cerium (IV): Synthesis, Reactivity, and Electrochemistry. Inorg. Chem..

[38] Salehisaki M., Rad N.E., Deacon G.B., Wang J., Guo Z., Junk P.C. (2022). Synthesis and Reactivity of Rare-Earth-N,N’-(Diphenyl) Formamidinate and Rare-Earth-N,N’-Bis(2,4-Dimethylphenyl) Formamidinate Complexes. Inorg. Chim. Acta..

[39] Deacon G.B., Phillips R.J. (1980). Relationships between the Carbon-Oxygen Stretching Frequencies of Carboxylato Complexes and the Type of Carboxylate Coordination. Coord. Chem. Rev..

[40] Chesman A.S.R., Turner D.R., Moubaraki B., Murray K.S., Deacon G.B., Batten S.R. (2009). Lanthaballs: Chiral, Structurally Layered Polycarbonate Tridecanuclear Lanthanoid Clusters. Chem. A Eur. J..

[41] Chesman A.S.R., Turner D.R., Moubaraki B., Murray K.S., Deacon G.B., Batten S.R. (2012). Tetradecanuclear Polycarbonatolanthanoid Clusters: Diverse Coordination Modes of Carbonate Providing Access to Novel Core Geometries. Dalton Trans..

[42] Vallina B., Rodriguez-Blanco J.D., Brown A.P., Blanco J.A., Benning L.G. (2013). Amorphous Dysprosium Carbonate: Characterization, Stability, and Crystallization Pathways. J. Nanopart. Res..

[43] Deacon G.B. (1970). Syntheses of Organometallic Compounds by Thermal Decarboxylation. Organomet. Chem. Rev. Sect. A.

[44] Deacon G.B., Faulks S.J., Pain G.N. (1986). The Synthesis of Organometallics by Decarboxylation Reactions. Adv. Organomet. Chem..

[45] Shannon R.D. (1976). Revised effective ionic radii and systematic studies of interatomic distances in halides and chalcogenides. Acta Cryst..

[46] Blin F., Leary S.G., Wilson K., Deacon G., Junk P., Forsyth M. (2004). Corrosion Mitigation of Mild Steel by New Rare Earth Cinnamate Compounds. J. Appl. Electrochem..

[47] MacRae C.F., Sovago I., Cottrell S.J., Galek P.T.A., McCabe P., Pidcock E., Platings M., Shields G.P., Stevens J.S., Towler M. (2020). Mercury 4.0: From Visualization to Analysis, Design and Prediction. J. Appl. Crystallogr..

[48] McPhillips T.M., McPhillips S.E., Chiu H.J., Cohen A.E., Deacon A.M., Ellis P.J., Garman E., Gonzalez A., Sauter N.K., Phizackerley R.P. (2002). Blu-Ice and the Distributed Control System: Software for Data Acquisition and Instrument Control at Macromolecular Crystallography Beamlines. J. Synchrotron Radiat..

[49] Kabsch W. (1993). Automatic Processing of Rotation Diffraction Data from Crystals of Initially Unknown Symmetry and Cell Constants. J. Appl. Crystallogr..

[50] Sheldrick G.M. (2015). Crystal structure refinement with *SHELXL*. Acta Crystallogr. Sect. C Struct. Chem..

[51] Dolomanov O.V., Bourhis L.J., Gildea R.J., Howard J.A.K., Puschmann H. (2009). OLEX2: A Complete Structure Solution, Refinement and Analysis Program. J. Appl. Crystallogr..

[52] American Society for Testing and Materials (2004). American-Standard-Test-Methods in ASTM G31-72.

[53] Cowieson N.P., Aragao D., Clift M., Ericsson D., Gee C., Harrop S.J., Mudie N., Panjikar S., Price J., Riboldi-Tunnicliffe A. (2015). MX1: A bending-magnet crystallography beamline serving both chemical and macromolecular crystallography communities at the Australian Synchrotron. J. Synchrotron Radiat..

