# Supplementary material for: RE(III) 3-Furoate Complexes: Synthesis, Structure, and Corrosion Inhibiting Properties
Source: Molecules. 2022 Dec 13;27(24):8836. doi: 10.3390/molecules27248836 (PMC9784407; doi:10.3390/molecules27248836)
Supplement: Supplementary file 1 [file molecules-27-08836-s001.zip › molecules-2092852-supplementary.pdf]

Supplementary Material

RE(III) 3-furoate complexes: Synthesis, structure, and corrosion inhibiting properties

Vidushi P. Vithana [1], Zhifang Guo [1], Glen B. Deacon [2], Anthony E. Somers [3] and Peter C. Junk [1,*]

[1] College of Science & Engineering, James Cook University, Townsville, QLD 4811, Australia
[2] School of Chemistry, Monash University, Clayton, VIC 3800, Australia
[3] Institute for Frontier Materials, Deakin University, Burwood, VIC 3125, Australia
* Correspondence: peter.junk@jcu.edu.au

Content:

1. **Table S1:** Crystal data and structural refinement for RE 3-furoate complexes
2. **Table S2:** Selected bond lengths and RE…RE distances (Å) for the isostructural $[RE(3fur)_3(H_2O)_2]_n$ **1RE** series
3. **Table S3.** Selected bond angles (°) for the isostructural $[RE(3fur)_3(H_2O)_2]_n$ **1RE** series.
4. **Table S4**: Hydrogen bonds for $[Nd(3fur)_3(H_2O)_2]_n$ (**1Nd)** [d/Å and </°]
5. **Table S5**: Selected bond lengths and Na…RE distances (Å) for the isostructural $[NaRE(3fur)_4]_n$ **2RE** series
6. **Table S6.** Selected bond angles (°) for the isostructural $[NaRE(3fur)_4]_n$ **2RE** series.
7. **Figure S1:** IR spectra of $[RE(3fur)_3(H_2O)_2]_n$ **1RE** series
8. **Figure S2:** IR spectra of $[NaRE(3fur)_4]_n$ **2RE** series
9. **Figure S3:** TGA plots of the $[RE(3fur)_3(H_2O)_2]_n$ **1RE** series
10. **Figure S4:** TGA plots of the $[NaRE(3fur)_4]_n$ **2RE** series
11. **Figure S5:** IR spectra of $Ce_2(CO_3)_2$ from thermal decomposition of **1Ce**, reported $Dy_2(CO_3)_3{\cdot}4H_2O$ (middle) and $Na_3Yb(CO_3)_3$ (right) from thermal decomposition of **2Yb**
12. **Figure S6:** Mild steel coupons immersed in control and the three best inhibited solutions for 168 h (Left – Trial 1; right – Trial 2.)

Table S1. Crystal data and structural refinement for rare earth 3-furoate complexes

	1La	1Ce	1Pr	1Nd	1Gd	1Dy	1Ho
	$[La(3fur)(H_2O)_2]_n$	$[Ce(3fur)(H_2O)_2]_n$	$[Pr(3fur)(H_2O)_2]_n$	$[Nd(3fur)(H_2O)_2]_n$	$[Gd(3fur)(H_2O)_2]_n$	$[Dy(3fur)(H_2O)_2]_n$	$[Ho(3fur)(H_2O)_2]_n$
Formula	$C_{15}H_{13}LaO_{11}$	$C_{15}H_{13}CeO_{11}$	$C_{15}H_{13}O_{11}Pr$	$C_{15}H_{13}NdO_{11}$	$C_{15}H_{13}GdO_{11}$	$C_{15}H_{13}DyO_{11}$	$C_{15}H_{13}HoO_{11}$
M_r	508.16	509.37	510.16	513.49	526.50	531.75	534.18
Space group	C2/c	C2/c	C2/c	C2/c	C2/c	C2/c	C2/c
a (Å)	11.500(2)	11.513(2)	11.533(2)	11.547(2)	11.493(2)	11.461(2)	11.434(2)
b (Å)	27.339(6)	27.247(5)	27.173(5)	27.114(5)	26.991(5)	26.918(5)	26.886(5)
c (Å)	6.0950(12)	6.0820(12)	6.0720(12)	6.0560(12)	6.0220(12)	6.0140(12)	5.9990(12)
α (°)	90	90	90	90	90	90	90
β (°)	117.29(3)	117.75(3)	118.04(3)	118.27(3)	118.65(3)	118.85(3)	118.89(3)
γ (°)	90	90	90	90	90	90	90
V (Å^3)	1703.0(7)	1688.4(7)	1679.5(7)	1669.9(7)	1639.4(7)	1625.2(7)	1614.8(7)
Z	4	4	4	4	4	4	4
ρ_{calc}, g cm^{-3}	1.982	2.044	2.018	2.042	2.133	2.173	2.197
μ, mm^{-1}	2.569	2.757	2.962	3.171	4.108	4.661	4.964
N_τ	9803	27366	9244	18672	18366	8857	8562
N (R_{int})	1473 (0.0389)	1507(0.0634)	1449(0.0360)	1462(0.0251)	1386(0.0267)	1396(0.0382)	1407(0.0753)
R_1(I > 2σ(I))	0.0230	0.0240	0.0179	0.0136	0.0156	0.0207	0.0379
wR_2 (all data)	0.0612	0.0616	0.0468	0.0323	0.0410	0.0524	0.0991
GOF	1.048	1.087	1.097	1.158	1.095	1.073	1.035

	1Y	2Ho	2Y	2Er	2Yb	2Lu
	**$[Y(3fur)(H_2O)_2]_n$**	**$[NaHo(3fur)_4]_n$**	**$[NaY(3fur)_4]_n$**	**$[NaEr(3fur)_4]_n$**	**$[NaYb(3fur)_4]_n$**	**$[NaLu(3fur)_4]_n$**
Formula	$C_{15}H_{13}O_{11}Y$	$C_{20}H_{12}HoNaO_{12}$	$C_{20}H_{12}NaO_{12}Y$	$C_{20}H_{12}ErNaO_{12}$	$C_{20}H_{12}NaO_{12}Yb$	$C_{20}H_{12}LuNaO_{12}$
M_r	458.16	632.22	556.20	634.55	640.33	642.26
Space group	C2/c	P-4n2	P-4n2	P-4n2	P-4n2	P-4n2
a (Å)	11.446(2)	14.257(2)	14.240(2)	14.2457(2)	14.184(2)	14.177(2)
b (Å)	26.889(5)	14.257(2)	14.240(2)	14.2457(2)	14.184(2)	14.177(2)
c (Å)	6.0030(12)	21.587(4)	21.691(4)	21.5746(5)	21.501(4)	21.541(4)
α (°)	90	90	90	90	90	90
β (°)	118.84(3)	90	90	90	90	90
γ (°)	90	90	90	90	90	90
V (Å^3)	1618.4(7)	4387.8(15)	4398.4(15)	4378.35(16)	4326.0(15)	4329.5(15)
Z	4	8	8	8	8	8
ρ_{calc}, g cm^{-3}	1.880	1.914	1.680	1.925	1.966	1.971
μ, mm^{-1}	3.667	3.691	2.736	3.918	4.409	4.646
N_τ	18244	50797	53087	28175	49021	50096
N (R_{int})	1417(0.0501)	4167(0.0478)	4496(0.0565)	6598(0.0738)	3815(0.0390)	4128(0.0521)
R_1($I > 2\sigma(I)$)	0.0201	0.0201	0.0296	0.0377	0.0129	0.0202
wR_2 (all data)	0.0544	0.0549	0.0817	0.0934	0.0358	0.0522
GOF	1.077	1.068	1.043	1.047	1.095	1.090

Table S2. Selected bond lengths and RE…RE distances (Å) for the isostructural $[RE(3fur)_3(H_2O)_2]_n$ **1RE** series.

	La1 **(1La)**	Ce1 **(1Ce)**	Pr1 **(1Pr)**	Nd1 **(1Nd)**	Gd1 **(1Gd)**	Dy1 **(1Dy)**	Ho1 **(1Ho)**	Y1 **(1Y)**
RE1 =RE1#1	5.1559(18)	5.1374(18)	5.1306(18)	5.1246(18)	5.0823(18)	5.0582(18)	5.0451(18)	5.0528(18)
O1#4	2.410(2)	2.385(2)	2.3624(18)	2.3487(15)	2.2968(19)	2.267(2)	2.252(4)	2.2535(14)
O1	2.410(2)	2.385(2)	2.3624(18)	2.3487(15)	2.2968(19)	2.267(2)	2.252(4)	2.2535(14)
O2#1	2.459(2)	2.4306(19)	2.4139(16)	2.3991(13)	2.3443(17)	2.308(2)	2.294(4)	2.2969(12)
O2#3	2.459(2)	2.4306(19)	2.4139(16)	2.3991(13)	2.3443(17)	2.308(2)	2.294(4)	2.2969(12)
O5#4	2.538(2)	2.513(2)	2.5010(17)	2.4848(14)	2.4464(18)	2.436(2)	2.422(4)	2.4196(14)
O5	2.538(2)	2.513(2)	2.5009(17)	2.4848(14)	2.4464(18)	2.436(2)	2.422(4)	2.4197(14)
O4#4	2.640(2)	2.621 (19)	2.6026(17)	2.5830(14)	2.5276(18)	2.506(2)	2.494(4)	2.4908(13)
O4	2.640(2))	2.621 (19)	2.6026(17)	2.5830(14)	2.5276(18)	2.506(2)	2.494(4)	2.4908(13)

Table S3. Selected bond angles (°) for the isostructural $[RE(3fur)_3(H_2O)_2]_n$ **1RE** series.

	**(1La)**	**(1Ce)**	**(1Pr)**	**(1Nd)**	**(1Gd)**	**(1Dy)**	**(1Ho)**	**(1Y)**
O4-Nd-O4#4	126.59(10)	126.72(9)	126.91(7)	127.16(6)	127.28(9)	127.46(10)	127.36(18)	127.62(6)
Nd1#1-Nd1-Nd1#3	167.764(13)	167.565(13)	167.485(13)	167.339(5)	166.961(13)	166.873(13)	166.706(13)	166.725(13)

Table S4. Hydrogen bonds for $[Nd(3fur)_3(H_2O)_2]_n$ (**1Nd**) [d/Å and </°].

D-H…A	d(D-H)	d(H…A)	d(D…A)	<(DHA)
O4#1-H4A#1…O5#4	0.8534(14)	2.1716(15)	2.954(2)	152.32(10)
O4#3-H4A#3…O5	0.8534(14)	2.1716(15)	2.954(2)	152.32(10)

Table S5. Selected bond lengths and Na…RE distances (Å) for the isostructural $[NaRE(3fur)_4]_n$ **2RE** series.

	Ho1 (**2Ho**)	Y1 (**2Y**)	Er1 (**2Er**)	Yb1 (**2Yb**)	Lu1 (**2Lu**)
Na1	3.403(2)	3.4031(15)	3.401(3)	3.3841(16)	3.381(2)
O1	2.392(4)	2.384(3)	2.372(5)	2.360(3)	2.351(4)
O1#1	2.392(4)	2.384(3)	2.372(5)	2.360(3)	2.351(4)
O2	2.453(4)	2.457(3)	2.449(6)	2.432(3)	2.428(4)
O2#1	2.453(4)	2.457(3)	2.449(6)	2.432(3)	2.428(4)
O4	2.238(4)	2.239(3)	2.221(5)	2.208(3)	2.196(4)
O4#1	2.238(4)	2.238(3)	2.222(5)	2.208(3)	2.196(4)
O5#2	2.357(3)	2.356(2)	2.340(5)	2.321(3)	2.313(4)
O5#3	2.357(3)	2.356(2)	2.340(5)	2.321(3)	2.313(4)

	Ho2 (**2Ho**)	Y2 (**2Y**)	Er2 (**2Er**)	Yb2 (**2Yb**)	Lu2 (**2Lu**)
Na1	3.398(2)	3.4005(15)	3.392(3)	3.3705(16)	3.374(2)
O7	2.356(3)	2.355(2)	2.340(5)	2.317(3)	2.312(4)
O7#4	2.355(3)	2.355(2)	2.340(5)	2.317(3)	2.311(4)
O8	2.510(4)	2.507(3)	2.509(6)	2.495(3)	2.492(4)
O8#4	2.510(4)	2.507(3)	2.509(6)	2.495(3)	2.492(4)
O10	2.212(4)	2.213(3)	2.207(5)	2.180(3)	2.170(4)
O10#4	2.212(4)	2.213(3)	2.207(5)	2.180(3)	2.170(4)
O11#5	2.391(4)	2.390(3)	2.367(5)	2.349(3)	2.341(4)
O11#6	2.391(4)	2.390(3)	2.367(5)	2.349(3)	2.341(4)

	Na1 (**2Ho**)	Na1 (**2Y**)	Na1 (**2Er**)	Na1 (**2Yb**)	Na1 (**2Lu**)
O1	2.323(4)	2.320(3)	2.327(6)	2.317(3)	2.310(5)
O2#1	2.347(4)	2.348(3)	2.347(6)	2.338(4)	2.334(5)
O5#2	2.418(4)	2.420(3)	2.429(6)	2.428(4)	2.430(5)
O7	2.344(5)	2.340(3)	2.344(7)	2.332(4)	2.333(5)

O8#4	2.388(4)	2.387(3)	2.389(6)	2.369(4)	2.374(5)
O11#6	2.367(4)	2.373(3)	2.368(6)	2.367(4)	2.368(5)

Table S6. Selected bond angles (°) for the isostructural $[NaRE(3fur)_4]_n$ **2RE** series.

	(2Ho)	(2Y)	(2Er)	(2Yb)	(2Lu)
C1-Yb1-C1#1	85.3(2)	85.18(15)	85.2(3)	85.81(9)	85.7(2)
C11-Yb2-C11#4	82.3(2)	82.48(15)	82.7(3)	82.54(18)	83.1(2)
Yb1-Na1-Yb2	174.29(7)	174.32(5)	174.54910)	175.26(7)	175.50(8)

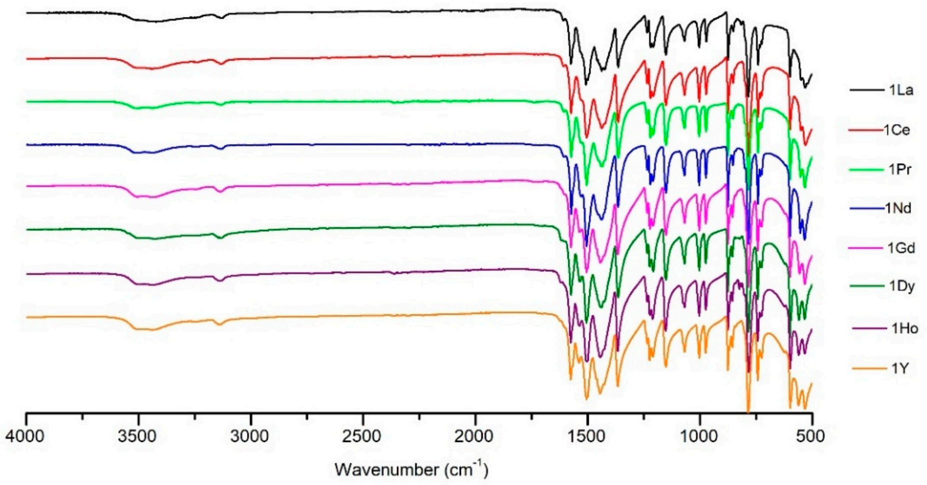

Figure S1. IR spectra of $[RE(3fur)_3(H_2O)_2]_n$ **1RE** series

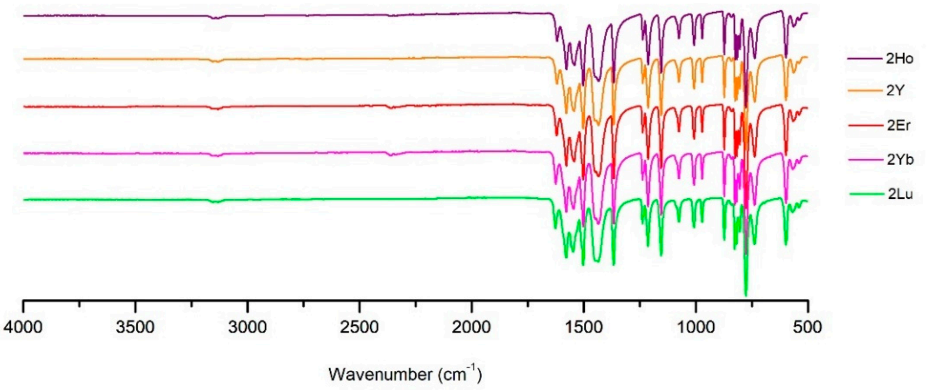

Figure S2. IR spectra of $[NaRE(3fur)_4]_n$ **2RE** series

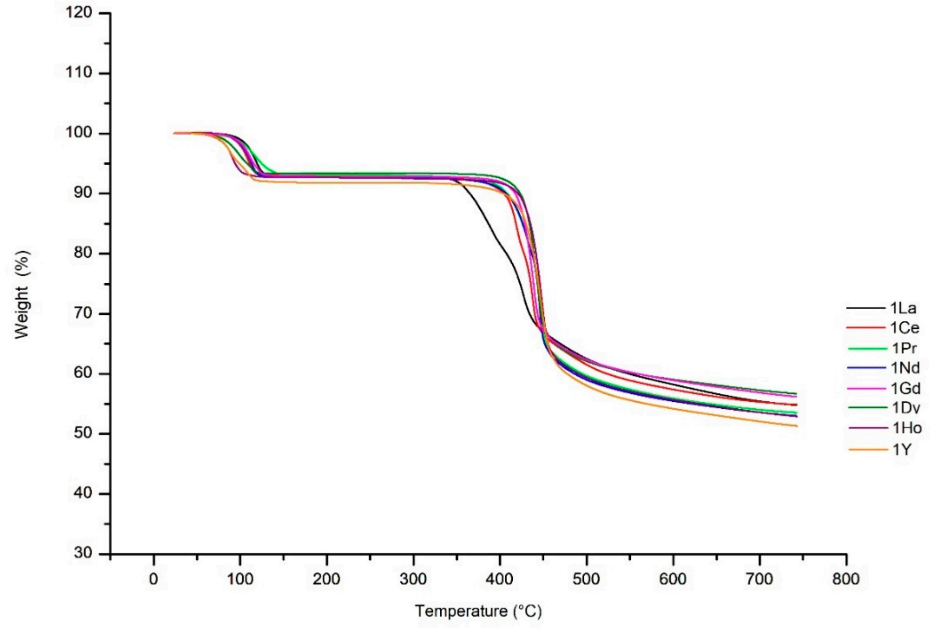

Figure S3. TGA plots of the $[RE(3fur)_3(H_2O)_2]_n$ **1RE** series

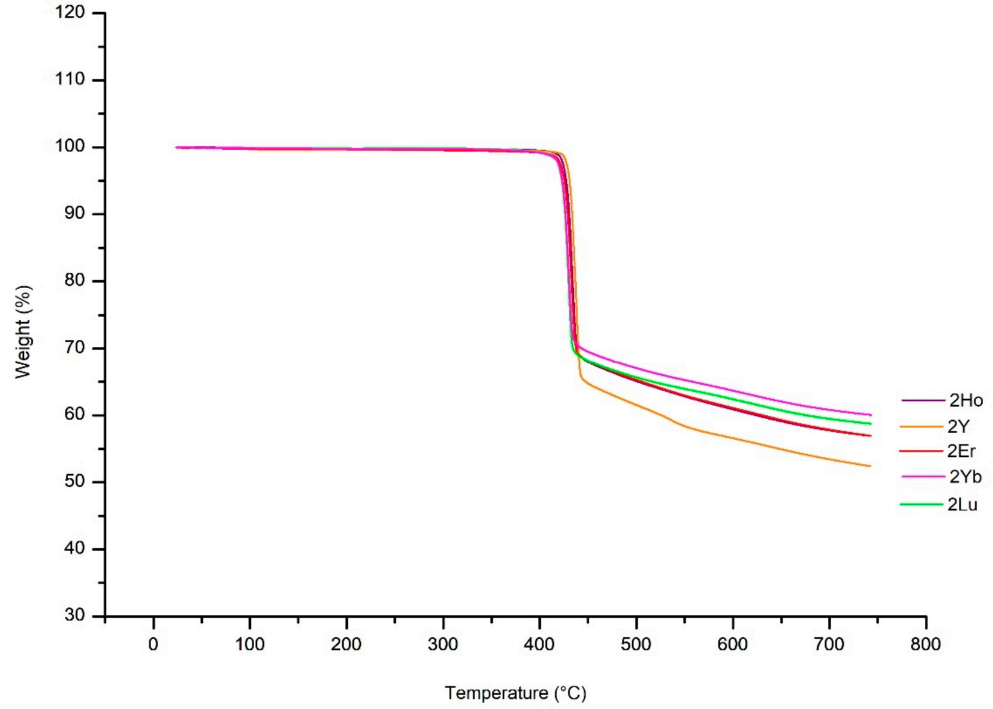

Figure S4. TGA plots of the $[NaRE(3fur)_4]_n$ **2RE** series

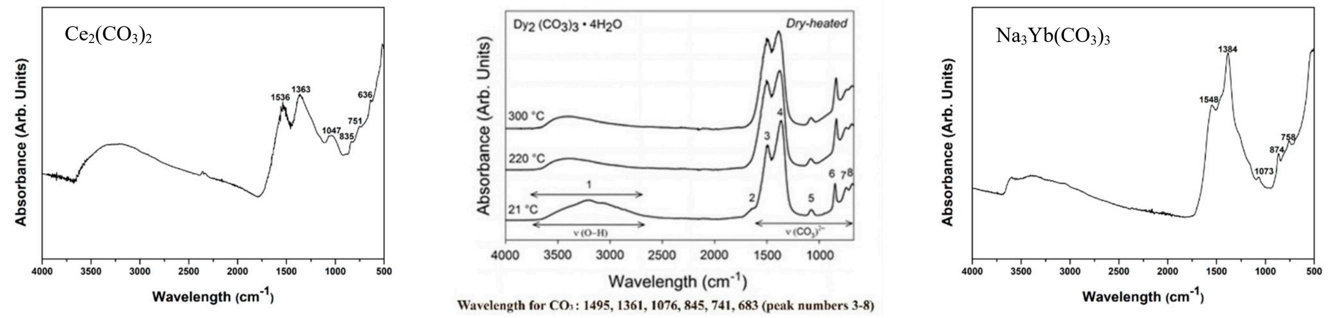

Figure S5. IR spectra of $Ce_2(CO_3)_2$ (Left) from thermal decomposition of **1Ce**, reported $Dy_2(CO_3)_3{\cdot}4H_2O$ (middle) [32] and $Na_3Yb(CO_3)_3$ (right) from thermal decomposition of **2Yb**

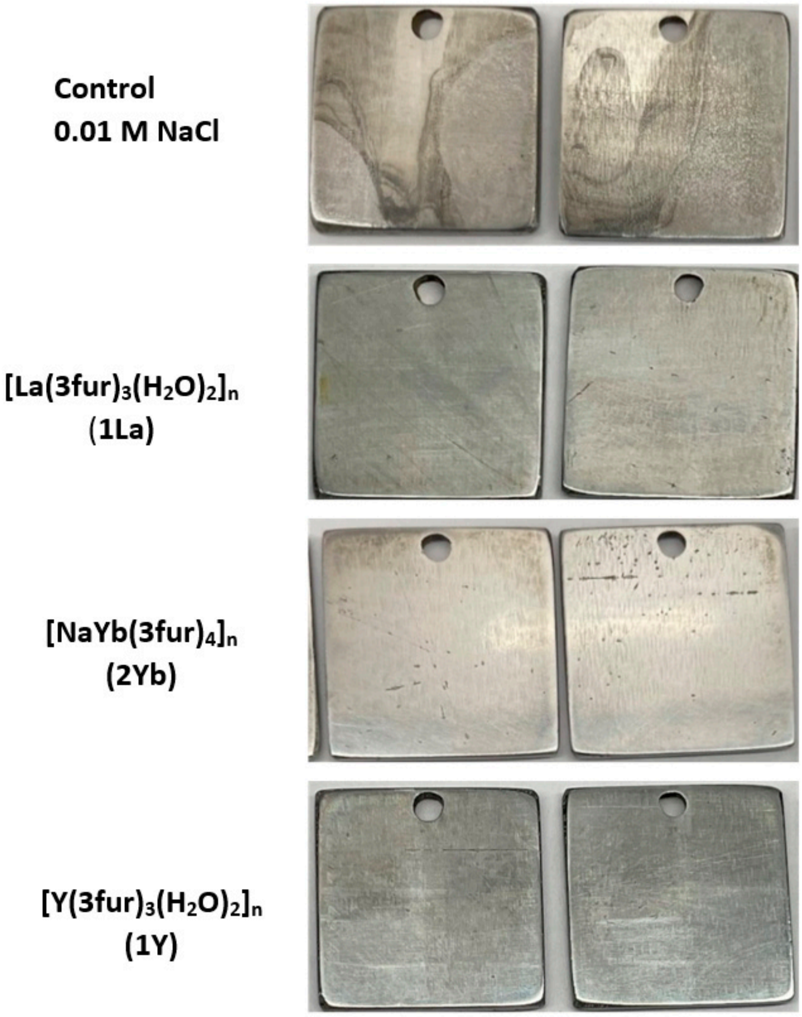

Figure S6. Mild steel coupons immersed in control and the three best inhibited solutions for 168 h (Left – Trial 1; right – Trial 2.)
