# Supplementary material for: RE(III) 3-Furoate Complexes: Synthesis, Structure, and Corrosion Inhibiting Properties
Source: Molecules. 2022 Dec 13;27(24):8836. doi: 10.3390/molecules27248836 (PMC9784407; doi:10.3390/molecules27248836)

checkCIF/PLATON report

Structure factors have been supplied for datablock(s) mx118_21_auto

THIS REPORT IS FOR GUIDANCE ONLY. IF USED AS PART OF A REVIEW PROCEDURE FOR PUBLICATION, IT SHOULD NOT REPLACE THE EXPERTISE OF AN EXPERIENCED CRYSTALLOGRAPHIC REFEREE.

No syntax errors found. CIF dictionary Interpreting this report

Datablock: mx118_21_auto

Bond precision: C-C = 0.0121 A Wavelength=0.71073

Cell:	a=14.2457(2)	b=14.2457(2)	c=21.5746(5)
	alpha=90	beta=90	gamma=90
Temperature:	123 K		

	Calculated	Reported
Volume	4378.35(16)	4378.35(16)
Space group	P -4 n 2	P -4 n 2
Hall group	P -4 -2n	P -4 -2n
Moiety formula	C20 H12 Er Na O12	C20 H12 Er Na O12
Sum formula	C20 H12 Er Na O12	C20 H12 Er Na O12
Mr	634.55	634.55
Dx,g cm-3	1.925	1.925
Z	8	8
Mu (mm-1)	3.918	3.918
F000	2456.0	2456.0
F000’	2455.58	
h,k,lmax	21,21,32	19,19,30
Nref	7721[4142]	6598
Tmin,Tmax	0.391,0.375	0.320,1.000
Tmin’	0.361	

Correction method= # Reported T Limits: Tmin=0.320 Tmax=1.000
AbsCorr = MULTI-SCAN

Data completeness= 1.59/0.85 Theta(max)= 32.158

R(reflections)= 0.0377(5639) wR2(reflections)= 0.0934(6598)

S = 1.047 Npar= 308

The following ALERTS were generated. Each ALERT has the format
test-name_ALERT_alert-type_alert-level.
Click on the hyperlinks for more details of the test.

Alert level C

```
PLAT213_ALERT_2_C Atom C4              has ADP max/min Ratio .....         3.7 prolat
PLAT213_ALERT_2_C Atom C14             has ADP max/min Ratio .....         3.7 prolat
PLAT220_ALERT_2_C NonSolvent   Resd 1  C   Ueq(max)/Ueq(min) Range         4.2 Ratio
PLAT220_ALERT_2_C NonSolvent   Resd 1  O   Ueq(max)/Ueq(min) Range         4.3 Ratio
PLAT241_ALERT_2_C High   'MainMol' Ueq as Compared to Neighbors of          O3 Check
PLAT241_ALERT_2_C High   'MainMol' Ueq as Compared to Neighbors of          O6 Check
PLAT241_ALERT_2_C High   'MainMol' Ueq as Compared to Neighbors of          O9 Check
PLAT342_ALERT_3_C Low Bond Precision on  C-C Bonds ..............     0.01206 Ang.
PLAT601_ALERT_2_C Unit Cell Contains Solvent Accessible VOIDS of .          39 Ang**3
PLAT910_ALERT_3_C Missing # of FCF Reflection(s) Below Theta(Min).           8 Note
PLAT971_ALERT_2_C Check Calcd Resid. Dens.  1.73A   From C6              1.55 eA-3
PLAT971_ALERT_2_C Check Calcd Resid. Dens.  1.17A   From Er2             1.51 eA-3
PLAT972_ALERT_2_C Check Calcd Resid. Dens.  0.66A   From Er2            -1.77 eA-3
PLAT972_ALERT_2_C Check Calcd Resid. Dens.  0.72A   From Er1            -1.70 eA-3
PLAT972_ALERT_2_C Check Calcd Resid. Dens.  0.68A   From Er2            -1.60 eA-3
PLAT973_ALERT_2_C Check Calcd Positive Resid. Density on       Er2       1.21 eA-3
PLAT973_ALERT_2_C Check Calcd Positive Resid. Density on       Er1       1.14 eA-3
PLAT975_ALERT_2_C Check Calcd Resid. Dens.  1.03A   From O11             0.92 eA-3
PLAT975_ALERT_2_C Check Calcd Resid. Dens.  0.76A   From O2              0.84 eA-3
PLAT977_ALERT_2_C Check Negative Difference Density on H20              -0.39 eA-3
```

Alert level G

```
PLAT003_ALERT_2_G Number of Uiso or Uij Restrained non-H Atoms ...           2 Report
PLAT004_ALERT_5_G Polymeric Structure Found with Maximum Dimension           3 Info
PLAT177_ALERT_4_G The CIF-Embedded .res File Contains DELU Records           1 Report
PLAT178_ALERT_4_G The CIF-Embedded .res File Contains SIMU Records           1 Report
PLAT398_ALERT_2_G Deviating  C-O-C    Angle From 120 for O3             108.1 Degree
PLAT398_ALERT_2_G Deviating  C-O-C    Angle From 120 for O6             106.0 Degree
PLAT398_ALERT_2_G Deviating  C-O-C    Angle From 120 for O9             109.4 Degree
PLAT398_ALERT_2_G Deviating  C-O-C    Angle From 120 for O12            107.4 Degree
PLAT794_ALERT_5_G Tentative Bond Valency for Er1       (III)     .       3.01 Info
PLAT794_ALERT_5_G Tentative Bond Valency for Er2       (III)     .       3.07 Info
PLAT860_ALERT_3_G Number of Least-Squares Restraints ............           7 Note
PLAT912_ALERT_4_G Missing # of FCF Reflections Above STh/L=  0.600         314 Note
PLAT933_ALERT_2_G Number of OMIT Records in Embedded .res File ...           1 Note
PLAT950_ALERT_5_G Calculated (ThMax) and CIF-Reported Hmax Differ           2 Units
PLAT951_ALERT_5_G Calculated (ThMax) and CIF-Reported Kmax Differ           2 Units
PLAT952_ALERT_5_G Calculated (ThMax) and CIF-Reported Lmax Differ           2 Units
PLAT958_ALERT_1_G Calculated (ThMax) and Actual (FCF) Lmax Differ           2 Units
PLAT978_ALERT_2_G Number C-C Bonds with Positive Residual Density.           2 Info
```

0 **ALERT level A** = Most likely a serious problem - resolve or explain
0 **ALERT level B** = A potentially serious problem, consider carefully
20 **ALERT level C** = Check. Ensure it is not caused by an omission or oversight
18 **ALERT level G** = General information/check it is not something unexpected

1 ALERT type 1 CIF construction/syntax error, inconsistent or missing data

```
25 ALERT type 2 Indicator that the structure model may be wrong or deficient
 3 ALERT type 3 Indicator that the structure quality may be low
 3 ALERT type 4 Improvement, methodology, query or suggestion
 6 ALERT type 5 Informative message, check
```

It is advisable to attempt to resolve as many as possible of the alerts in all categories. Often the minor alerts point to easily fixed oversights, errors and omissions in your CIF or refinement strategy, so attention to these fine details can be worthwhile. In order to resolve some of the more serious problems it may be necessary to carry out additional measurements or structure refinements. However, the purpose of your study may justify the reported deviations and the more serious of these should normally be commented upon in the discussion or experimental section of a paper or in the "special_details" fields of the CIF. checkCIF was carefully designed to identify outliers and unusual parameters, but every test has its limitations and alerts that are not important in a particular case may appear. Conversely, the absence of alerts does not guarantee there are no aspects of the results needing attention. It is up to the individual to critically assess their own results and, if necessary, seek expert advice.

Publication of your CIF in IUCr journals

A basic structural check has been run on your CIF. These basic checks will be run on all CIFs submitted for publication in IUCr journals (*Acta Crystallographica*, *Journal of Applied Crystallography*, *Journal of Synchrotron Radiation*); however, if you intend to submit to *Acta Crystallographica Section C* or *E* or *IUCrData*, you should make sure that full publication checks are run on the final version of your CIF prior to submission.

Publication of your CIF in other journals

Please refer to the *Notes for Authors* of the relevant journal for any special instructions relating to CIF submission.

PLATON version of 13/07/2021; check.def file version of 13/07/2021

Datablock mx118_21_auto - ellipsoid plot

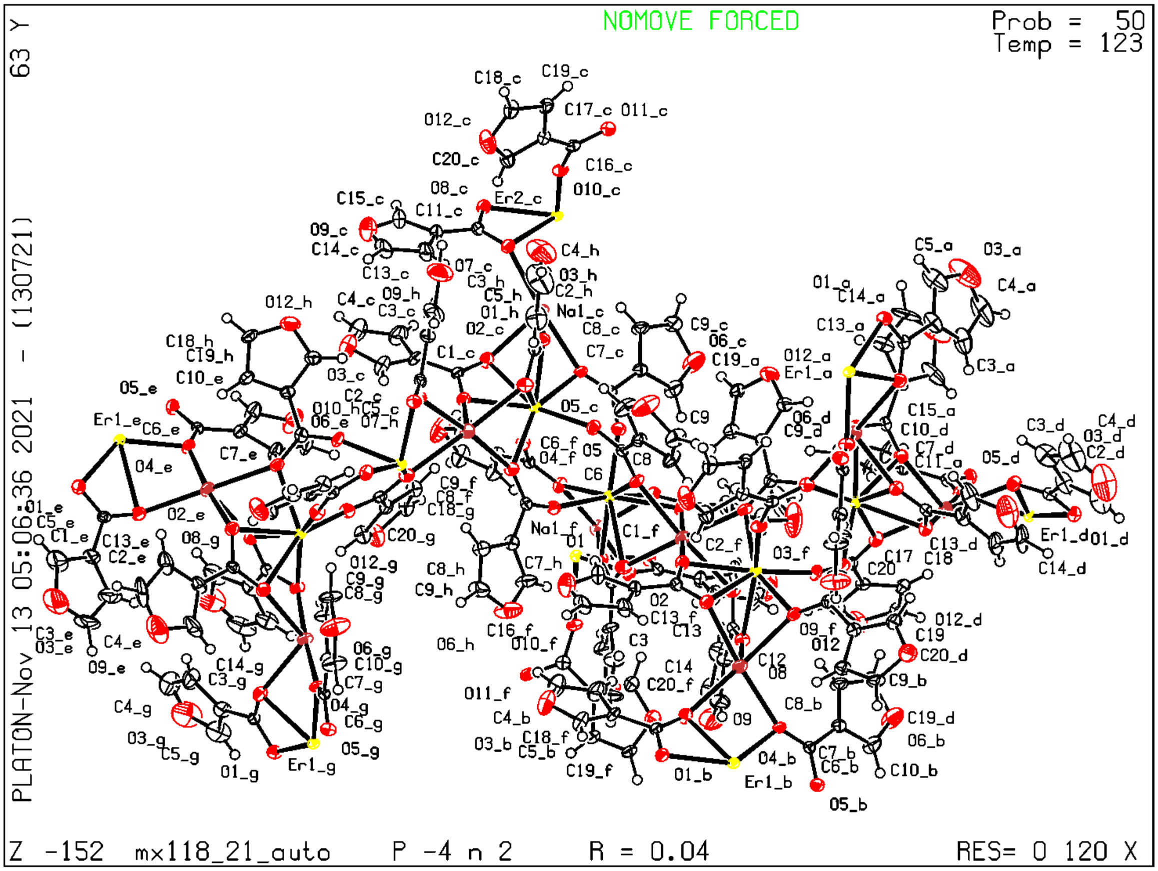

Supplement: Supplementary file 1 [file molecules-27-08836-s001.zip › xray files/11 checkcif.pdf]
