# Supplementary material for: RE(III) 3-Furoate Complexes: Synthesis, Structure, and Corrosion Inhibiting Properties
Source: Molecules. 2022 Dec 13;27(24):8836. doi: 10.3390/molecules27248836 (PMC9784407; doi:10.3390/molecules27248836)

# checkCIF/PLATON report

Structure factors have been supplied for datablock(s) vv3ce

THIS REPORT IS FOR GUIDANCE ONLY. IF USED AS PART OF A REVIEW PROCEDURE FOR PUBLICATION, IT SHOULD NOT REPLACE THE EXPERTISE OF AN EXPERIENCED CRYSTALLOGRAPHIC REFEREE.

No syntax errors found. CIF dictionary Interpreting this report

# Datablock: vv3ce

---

```
Bond precision:   C-C = 0.0048 A          Wavelength=0.71073

Cell:              a=11.513(2)     b=27.247(5)         c=6.0820(12)
                   alpha=90        beta=117.75(3)      gamma=90
Temperature:       100 K

                 Calculated                        Reported
Volume           1688.5(7)                         1688.4(7)
Space group      C 2/c                             C 1 2/c 1
Hall group       -C 2yc                            -C 2yc
Moiety formula   C15 H13 Ce O11                    C15 H13 Ce O11
Sum formula      C15 H13 Ce O11                    C15 H13 Ce O11
Mr               509.37                            509.37
Dx,g cm-3        2.004                             2.004
Z                4                                 4
Mu (mm-1)        2.757                             2.757
F000             996.0                             996.0
F000'            995.58
h,k,lmax         13,32,7                           13,32,7
Nref             1510                              1507
Tmin,Tmax        0.820,0.946                       0.334,0.432
Tmin'            0.759

Correction method= # Reported T Limits: Tmin=0.334 Tmax=0.432
AbsCorr = NONE

Data completeness= 0.998           Theta(max)= 25.040

                                                    wR2(reflections)=
R(reflections)= 0.0240( 1474)                       0.0616( 1507)

S = 1.087                    Npar= 143
```

The following ALERTS were generated. Each ALERT has the format
**test-name_ALERT_alert-type_alert-level.**
Click on the hyperlinks for more details of the test.

### Alert level C

```
PLAT057_ALERT_3_C Correction for Absorption Required   RT(exp) ...       1.15 Do !
PLAT094_ALERT_2_C Ratio of Maximum / Minimum Residual Density ....       3.11 Report
PLAT911_ALERT_3_C Missing FCF Refl Between Thmin & STh/L=    0.596          3 Report
PLAT971_ALERT_2_C Check Calcd Resid. Dens.  1.13Ang From Ce1             2.31 eA-3
PLAT976_ALERT_2_C Check Calcd Resid. Dens.  1.03Ang From O4       .     -0.50 eA-3
PLAT977_ALERT_2_C Check Negative Difference Density on H4A        .     -0.38 eA-3
```

### Alert level G

```
ABSMU01_ALERT_1_G  Calculation of _exptl_absorpt_correction_mu
                not performed for this radiation type.
PLAT003_ALERT_2_G Number of Uiso or Uij Restrained non-H Atoms ...          4 Report
PLAT004_ALERT_5_G Polymeric Structure Found with Maximum Dimension          2 Info
PLAT007_ALERT_5_G Number of Unrefined Donor-H Atoms .............           2 Report
PLAT128_ALERT_4_G Alternate Setting for Input Space Group     C2/c       I2/a Note
PLAT178_ALERT_4_G The CIF-Embedded .res File Contains SIMU Records          1 Report
PLAT300_ALERT_4_G Atom Site Occupancy of O6         Constrained at        0.5 Check
PLAT300_ALERT_4_G Atom Site Occupancy of C8         Constrained at        0.5 Check
PLAT300_ALERT_4_G Atom Site Occupancy of C9         Constrained at        0.5 Check
PLAT300_ALERT_4_G Atom Site Occupancy of C10        Constrained at        0.5 Check
PLAT300_ALERT_4_G Atom Site Occupancy of H8         Constrained at        0.5 Check
PLAT300_ALERT_4_G Atom Site Occupancy of H9         Constrained at        0.5 Check
PLAT300_ALERT_4_G Atom Site Occupancy of H10        Constrained at        0.5 Check
PLAT301_ALERT_3_G Main Residue  Disorder .............(Resd  1  )         14% Note
PLAT398_ALERT_2_G Deviating  C-O-C   Angle From 120 for O3        .     106.1 Degree
PLAT398_ALERT_2_G Deviating  C-O-C   Angle From 120 for O6        .     107.3 Degree
PLAT767_ALERT_4_G INS Embedded LIST 6 Instruction Should be LIST 4     Please Check
PLAT789_ALERT_4_G Atoms with Negative _atom_site_disorder_group  #          7 Check
PLAT794_ALERT_5_G Tentative Bond Valency for Ce1       (III)      .      3.32 Info
PLAT860_ALERT_3_G Number of Least-Squares Restraints ............          18 Note
PLAT883_ALERT_1_G No Info/Value for _atom_sites_solution_primary .     Please Do !
PLAT909_ALERT_3_G Percentage of I>2sig(I) Data at Theta(Max) Still        94% Note
PLAT933_ALERT_2_G Number of HKL-OMIT Records in Embedded .res File          2 Note
PLAT978_ALERT_2_G Number C-C Bonds with Positive Residual Density.          4 Info
```

0 **ALERT level A** = Most likely a serious problem - resolve or explain
0 **ALERT level B** = A potentially serious problem, consider carefully
6 **ALERT level C** = Check. Ensure it is not caused by an omission or oversight
24 **ALERT level G** = General information/check it is not something unexpected

2 ALERT type 1 CIF construction/syntax error, inconsistent or missing data
9 ALERT type 2 Indicator that the structure model may be wrong or deficient
5 ALERT type 3 Indicator that the structure quality may be low
11 ALERT type 4 Improvement, methodology, query or suggestion
3 ALERT type 5 Informative message, check

It is advisable to attempt to resolve as many as possible of the alerts in all categories. Often the minor alerts point to easily fixed oversights, errors and omissions in your CIF or refinement strategy, so attention to these fine details can be worthwhile. In order to resolve some of the more serious problems it may be necessary to carry out additional measurements or structure refinements. However, the purpose of your study may justify the reported deviations and the more serious of these should normally be commented upon in the discussion or experimental section of a paper or in the "special_details" fields of the CIF. checkCIF was carefully designed to identify outliers and unusual parameters, but every test has its limitations and alerts that are not important in a particular case may appear. Conversely, the absence of alerts does not guarantee there are no aspects of the results needing attention. It is up to the individual to critically assess their own results and, if necessary, seek expert advice.

**Publication of your CIF in IUCr journals**

A basic structural check has been run on your CIF. These basic checks will be run on all CIFs submitted for publication in IUCr journals (*Acta Crystallographica*, *Journal of Applied Crystallography*, *Journal of Synchrotron Radiation*); however, if you intend to submit to *Acta Crystallographica Section C* or *E* or *IUCrData*, you should make sure that full publication checks are run on the final version of your CIF prior to submission.

**Publication of your CIF in other journals**

Please refer to the *Notes for Authors* of the relevant journal for any special instructions relating to CIF submission.

---

**PLATON version of 12/09/2022; check.def file version of 09/08/2022**

**Datablock vv3ce** - ellipsoid plot

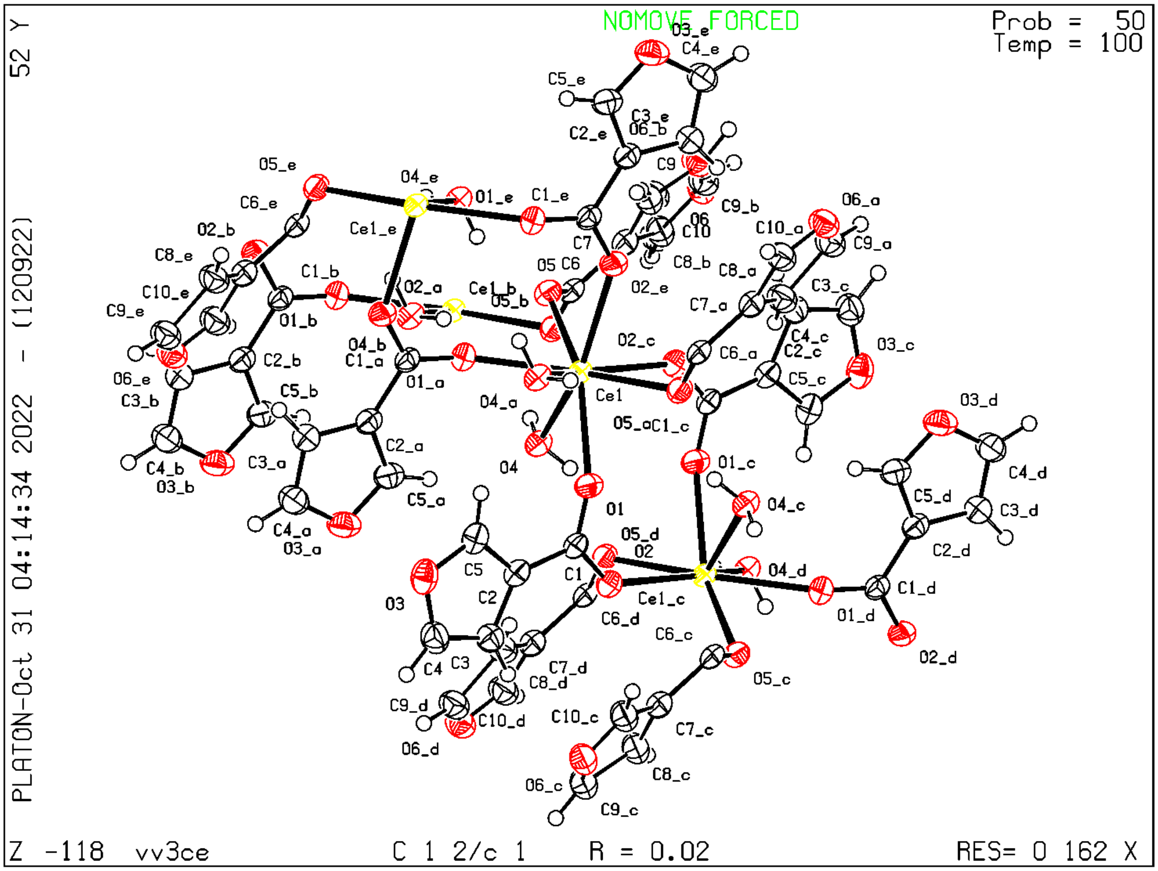

Supplement: Supplementary file 1 [file molecules-27-08836-s001.zip › xray files/2 checkcif.pdf]
