# Supplementary material for: RE(III) 3-Furoate Complexes: Synthesis, Structure, and Corrosion Inhibiting Properties
Source: Molecules. 2022 Dec 13;27(24):8836. doi: 10.3390/molecules27248836 (PMC9784407; doi:10.3390/molecules27248836)

checkCIF/PLATON report

Structure factors have been supplied for datablock(s) vv3ho

THIS REPORT IS FOR GUIDANCE ONLY. IF USED AS PART OF A REVIEW PROCEDURE FOR PUBLICATION, IT SHOULD NOT REPLACE THE EXPERTISE OF AN EXPERIENCED CRYSTALLOGRAPHIC REFEREE.

No syntax errors found. CIF dictionary Interpreting this report

Datablock: vv3ho

Bond precision: C-C = 0.0089 A Wavelength=0.71073

Cell:	a=14.257(2)	b=14.257(2)	c=21.587(4)
	alpha=90	beta=90	gamma=90
Temperature:	100 K		

	Calculated	Reported
Volume	4387.8(15)	4387.8(15)
Space group	P -4 n 2	P -4 n 2
Hall group	P -4 -2n	P -4 -2n
Moiety formula	C20 H12 Ho Na O12	C20 H12 Ho Na O12
Sum formula	C20 H12 Ho Na O12	C20 H12 Ho Na O12
Mr	632.22	632.22
Dx,g cm-3	1.914	1.914
Z	8	8
Mu (mm-1)	3.691	3.691
F000	2448.0	2448.0
F000'	2447.85	
h,k,lmax	17,17,26	17,16,25
Nref	4173[2272]	4167
Tmin,Tmax	0.801,0.895	0.381,0.432
Tmin'	0.691	

Correction method= # Reported T Limits: Tmin=0.381 Tmax=0.432
AbsCorr = MULTI-SCAN

Data completeness= 1.83/1.00 Theta(max)= 25.665

R(reflections)= 0.0201(4073) wR2(reflections)= 0.0549(4167)

S = 1.068 Npar= 308

The following ALERTS were generated. Each ALERT has the format
test-name_ALERT_alert-type_alert-level.
Click on the hyperlinks for more details of the test.

Alert level C

```
PLAT090_ALERT_3_C Poor Data / Parameter Ratio (Zmax > 18) ........       7.36 Note
PLAT220_ALERT_2_C NonSolvent   Resd 1  O   Ueq(max)/Ueq(min) Range        3.4 Ratio
PLAT241_ALERT_2_C High   'MainMol' Ueq as Compared to Neighbors of         O3 Check
PLAT241_ALERT_2_C High   'MainMol' Ueq as Compared to Neighbors of         O9 Check
PLAT241_ALERT_2_C High   'MainMol' Ueq as Compared to Neighbors of        O12 Check
PLAT342_ALERT_3_C Low Bond Precision on  C-C Bonds ...............    0.00888 Ang.
PLAT601_ALERT_2_C Unit Cell Contains Solvent Accessible VOIDS of .         39 Ang**3
PLAT975_ALERT_2_C Check Calcd Resid. Dens.  0.98Ang From O5      .       0.55 eA-3
```

Alert level G

```
ABSMU01_ALERT_1_G  Calculation of _exptl_absorpt_correction_mu
                   not performed for this radiation type.
PLAT003_ALERT_2_G Number of Uiso or Uij Restrained non-H Atoms ...          3 Report
PLAT004_ALERT_5_G Polymeric Structure Found with Maximum Dimension          3 Info
PLAT177_ALERT_4_G The CIF-Embedded .res File Contains DELU Records          2 Report
PLAT178_ALERT_4_G The CIF-Embedded .res File Contains SIMU Records          3 Report
PLAT398_ALERT_2_G Deviating  C-O-C   Angle From 120 for O3       .      108.0 Degree
PLAT398_ALERT_2_G Deviating  C-O-C   Angle From 120 for O6       .      106.7 Degree
PLAT398_ALERT_2_G Deviating  C-O-C   Angle From 120 for O9       .      107.2 Degree
PLAT398_ALERT_2_G Deviating  C-O-C   Angle From 120 for O12      .      105.5 Degree
PLAT779_ALERT_4_G Suspect or Irrelevant (Bond) Angle(s) in CIF ...       0.00 Deg.
              HO1  -O5   -HO1     3_566   1_555   4_656 ......     #   83 Check
PLAT779_ALERT_4_G Suspect or Irrelevant (Bond) Angle(s) in CIF ...       0.00 Deg.
              HO2  -O11  -HO2     7_456   1_555   2_675 ......     #   92 Check
PLAT794_ALERT_5_G Tentative Bond Valency for Ho1       (III)     .       3.31 Info
PLAT794_ALERT_5_G Tentative Bond Valency for Ho2       (III)     .       3.31 Info
PLAT860_ALERT_3_G Number of Least-Squares Restraints ............         14 Note
PLAT978_ALERT_2_G Number C-C Bonds with Positive Residual Density.          0 Info
```

0 **ALERT level A** = Most likely a serious problem - resolve or explain
0 **ALERT level B** = A potentially serious problem, consider carefully
8 **ALERT level C** = Check. Ensure it is not caused by an omission or oversight
15 **ALERT level G** = General information/check it is not something unexpected

1 ALERT type 1 CIF construction/syntax error, inconsistent or missing data
12 ALERT type 2 Indicator that the structure model may be wrong or deficient
3 ALERT type 3 Indicator that the structure quality may be low
4 ALERT type 4 Improvement, methodology, query or suggestion
3 ALERT type 5 Informative message, check

It is advisable to attempt to resolve as many as possible of the alerts in all categories. Often the minor alerts point to easily fixed oversights, errors and omissions in your CIF or refinement strategy, so attention to these fine details can be worthwhile. In order to resolve some of the more serious problems it may be necessary to carry out additional measurements or structure refinements. However, the purpose of your study may justify the reported deviations and the more serious of these should normally be commented upon in the discussion or experimental section of a paper or in the "special_details" fields of the CIF. checkCIF was carefully designed to identify outliers and unusual parameters, but every test has its limitations and alerts that are not important in a particular case may appear. Conversely, the absence of alerts does not guarantee there are no aspects of the results needing attention. It is up to the individual to critically assess their own results and, if necessary, seek expert advice.

Publication of your CIF in IUCr journals

A basic structural check has been run on your CIF. These basic checks will be run on all CIFs submitted for publication in IUCr journals (*Acta Crystallographica*, *Journal of Applied Crystallography*, *Journal of Synchrotron Radiation*); however, if you intend to submit to *Acta Crystallographica Section C* or *E* or *IUCrData*, you should make sure that full publication checks are run on the final version of your CIF prior to submission.

Publication of your CIF in other journals

Please refer to the *Notes for Authors* of the relevant journal for any special instructions relating to CIF submission.

PLATON version of 12/09/2022; check.def file version of 09/08/2022

Datablock vv3ho - ellipsoid plot

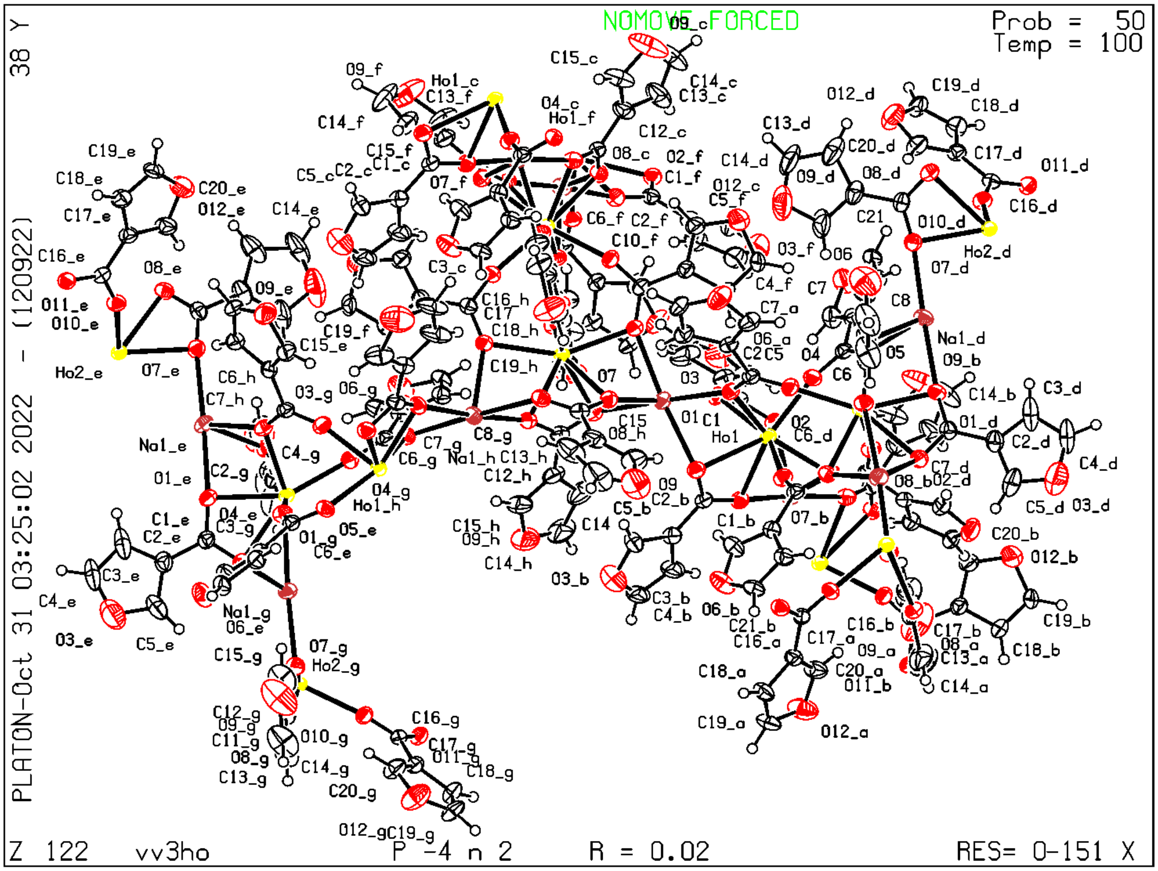

Supplement: Supplementary file 1 [file molecules-27-08836-s001.zip › xray files/9 checkcif.pdf]
