# Supplementary material for: RE(III) 3-Furoate Complexes: Synthesis, Structure, and Corrosion Inhibiting Properties
Source: Molecules. 2022 Dec 13;27(24):8836. doi: 10.3390/molecules27248836 (PMC9784407; doi:10.3390/molecules27248836)

checkCIF/PLATON report

Structure factors have been supplied for datablock(s) vv3ho

THIS REPORT IS FOR GUIDANCE ONLY. IF USED AS PART OF A REVIEW PROCEDURE FOR PUBLICATION, IT SHOULD NOT REPLACE THE EXPERTISE OF AN EXPERIENCED CRYSTALLOGRAPHIC REFEREE.

No syntax errors found. CIF dictionary Interpreting this report

Datablock: vv3ho

Bond precision:	C-C = 0.0092 A		Wavelength=0.71073
Cell:	a=11.434(2)	b=26.886(5)	c=5.9990(12)
	alpha=90	beta=118.89(3)	gamma=90
Temperature:	100 K		

	Calculated	Reported
Volume	1614.7(7)	1614.8(7)
Space group	C 2/c	C 1 2/c 1
Hall group	-C 2yc	-C 2yc
Moiety formula	C15 H13 Ho O11	C15 H13 Ho O11
Sum formula	C15 H13 Ho O11	C15 H13 Ho O11
Mr	534.18	534.18
Dx,g cm-3	2.197	2.197
Z	4	4
Mu (mm-1)	4.964	4.964
F000	1032.0	1032.0
F000'	1031.75	
h,k,lmax	13,32,7	13,32,7
Nref	1430	1407
Tmin,Tmax	0.706,0.905	0.217,0.432
Tmin'	0.603	

Correction method= # Reported T Limits: Tmin=0.217 Tmax=0.432
AbsCorr = MULTI-SCAN

Data completeness= 0.984 Theta(max)= 25.025

R(reflections)= 0.0379(1334) wR2(reflections)= 0.0991(1407)

S = 1.035 Npar= 144

The following ALERTS were generated. Each ALERT has the format
test-name_ALERT_alert-type_alert-level.
Click on the hyperlinks for more details of the test.

Alert level B

```
PLAT973_ALERT_2_B Check Calcd Positive Resid. Density on       Ho1       1.52 eA-3
```

Author Response: These are the ripples (residual density) features that are very closed to the heavy atom Ho and are typically more pronounced for heavier atoms.

Alert level C

```
PLAT088_ALERT_3_C Poor Data / Parameter Ratio ....................       9.77 Note
PLAT342_ALERT_3_C Low Bond Precision on  C-C Bonds ...............     0.0092 Ang.
PLAT911_ALERT_3_C Missing FCF Refl Between Thmin & STh/L=    0.595         25 Report
PLAT971_ALERT_2_C Check Calcd Resid. Dens.  0.92Ang From Ho1             1.88 eA-3
PLAT972_ALERT_2_C Check Calcd Resid. Dens.  1.12Ang From Ho1            -1.85 eA-3
PLAT972_ALERT_2_C Check Calcd Resid. Dens.  0.95Ang From Ho1            -1.67 eA-3
PLAT972_ALERT_2_C Check Calcd Resid. Dens.  0.87Ang From Ho1            -1.54 eA-3
PLAT976_ALERT_2_C Check Calcd Resid. Dens.  0.85Ang From O5      .      -0.62 eA-3
PLAT976_ALERT_2_C Check Calcd Resid. Dens.  0.86Ang From O4      .      -0.48 eA-3
PLAT976_ALERT_2_C Check Calcd Resid. Dens.  1.05Ang From O4      .      -0.47 eA-3
PLAT976_ALERT_2_C Check Calcd Resid. Dens.  0.86Ang From O1      .      -0.42 eA-3
```

Alert level G

```
ABSMU01_ALERT_1_G  Calculation of _exptl_absorpt_correction_mu
                not performed for this radiation type.
PLAT003_ALERT_2_G Number of Uiso or Uij Restrained non-H Atoms ...          4 Report
PLAT004_ALERT_5_G Polymeric Structure Found with Maximum Dimension          2 Info
PLAT007_ALERT_5_G Number of Unrefined Donor-H Atoms ..............          2 Report
PLAT128_ALERT_4_G Alternate Setting for Input Space Group     C2/c       I2/a Note
PLAT178_ALERT_4_G The CIF-Embedded .res File Contains SIMU Records          1 Report
PLAT300_ALERT_4_G Atom Site Occupancy of O6         Constrained at        0.5 Check
PLAT300_ALERT_4_G Atom Site Occupancy of C8         Constrained at        0.5 Check
PLAT300_ALERT_4_G Atom Site Occupancy of C9         Constrained at        0.5 Check
PLAT300_ALERT_4_G Atom Site Occupancy of C10        Constrained at        0.5 Check
PLAT300_ALERT_4_G Atom Site Occupancy of H8         Constrained at        0.5 Check
PLAT300_ALERT_4_G Atom Site Occupancy of H9         Constrained at        0.5 Check
PLAT300_ALERT_4_G Atom Site Occupancy of H10        Constrained at        0.5 Check
PLAT301_ALERT_3_G Main Residue  Disorder ..............(Resd  1  )        14% Note
PLAT398_ALERT_2_G Deviating  C-O-C   Angle From 120 for O3       .      106.0 Degree
PLAT398_ALERT_2_G Deviating  C-O-C   Angle From 120 for O6       .      107.6 Degree
PLAT767_ALERT_4_G INS Embedded LIST 6 Instruction Should be LIST 4     Please Check
PLAT789_ALERT_4_G Atoms with Negative _atom_site_disorder_group  #          7 Check
PLAT794_ALERT_5_G Tentative Bond Valency for Ho1       (III)     .       3.30 Info
PLAT860_ALERT_3_G Number of Least-Squares Restraints ............          18 Note
PLAT883_ALERT_1_G No Info/Value for _atom_sites_solution_primary .     Please Do !
PLAT909_ALERT_3_G Percentage of I>2sig(I) Data at Theta(Max) Still        91% Note
PLAT978_ALERT_2_G Number C-C Bonds with Positive Residual Density.          0 Info
```

```
 0 ALERT level A = Most likely a serious problem - resolve or explain
 1 ALERT level B = A potentially serious problem, consider carefully
11 ALERT level C = Check. Ensure it is not caused by an omission or oversight
23 ALERT level G = General information/check it is not something unexpected

 2 ALERT type 1 CIF construction/syntax error, inconsistent or missing data
13 ALERT type 2 Indicator that the structure model may be wrong or deficient
 6 ALERT type 3 Indicator that the structure quality may be low
11 ALERT type 4 Improvement, methodology, query or suggestion
 3 ALERT type 5 Informative message, check
```

It is advisable to attempt to resolve as many as possible of the alerts in all categories. Often the minor alerts point to easily fixed oversights, errors and omissions in your CIF or refinement strategy, so attention to these fine details can be worthwhile. In order to resolve some of the more serious problems it may be necessary to carry out additional measurements or structure refinements. However, the purpose of your study may justify the reported deviations and the more serious of these should normally be commented upon in the discussion or experimental section of a paper or in the "special_details" fields of the CIF. checkCIF was carefully designed to identify outliers and unusual parameters, but every test has its limitations and alerts that are not important in a particular case may appear. Conversely, the absence of alerts does not guarantee there are no aspects of the results needing attention. It is up to the individual to critically assess their own results and, if necessary, seek expert advice.

Publication of your CIF in IUCr journals

A basic structural check has been run on your CIF. These basic checks will be run on all CIFs submitted for publication in IUCr journals (*Acta Crystallographica*, *Journal of Applied Crystallography*, *Journal of Synchrotron Radiation*); however, if you intend to submit to *Acta Crystallographica Section C* or *E* or *IUCrData*, you should make sure that full publication checks are run on the final version of your CIF prior to submission.

Publication of your CIF in other journals

Please refer to the *Notes for Authors* of the relevant journal for any special instructions relating to CIF submission.

PLATON version of 18/05/2022; check.def file version of 17/05/2022

Datablock vv3ho - ellipsoid plot

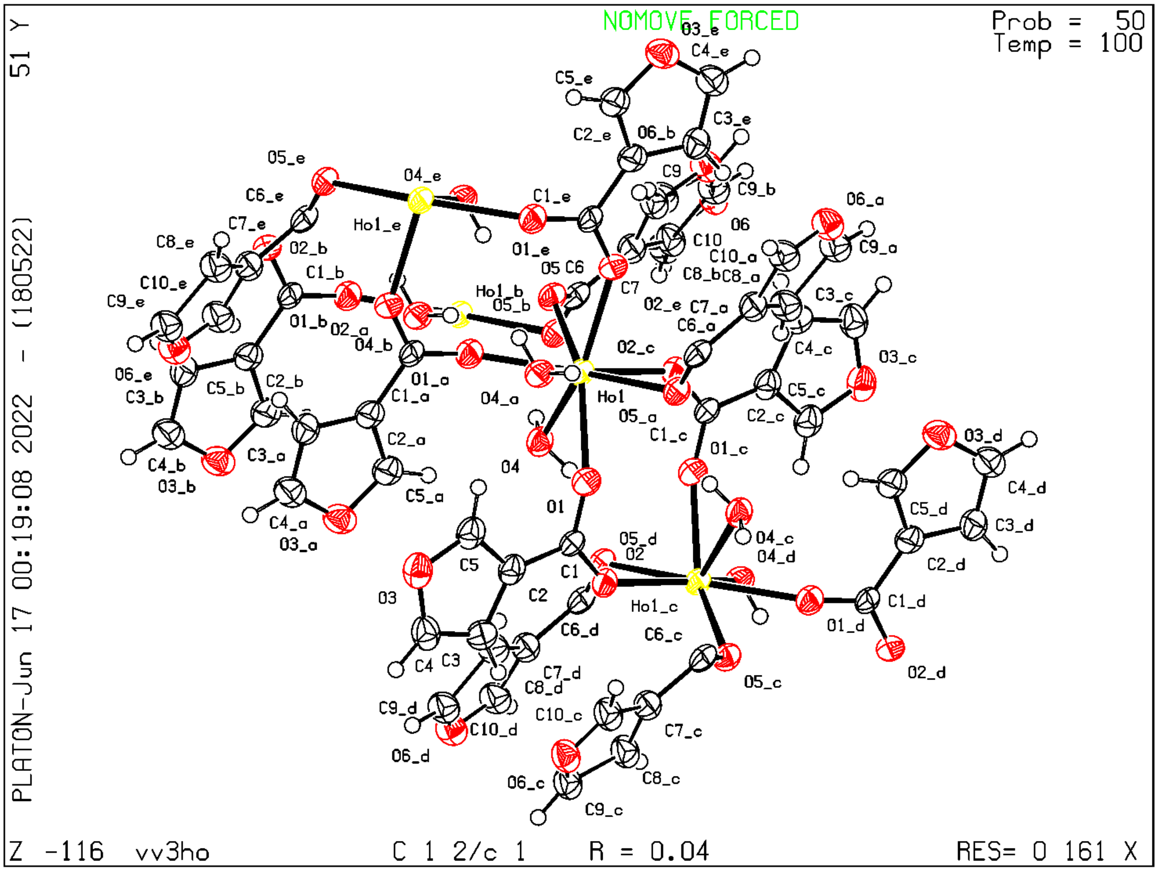

Supplement: Supplementary file 1 [file 7 checkcif.pdf › xray files/7 checkcif.pdf]
